# Targeted Inhibition of CD74^+^ Macrophages by Luteolin via CEBPB/P65 Signaling Ameliorates Osteoarthritis Progression

**DOI:** 10.1002/advs.202508472

**Published:** 2025-11-21

**Authors:** Rui Peng, Bo Yu, Lei Zhang, Zhaowen Xue, Lutian Yao, Qingjun Yang, Zitao Liu, Sizhi Wu, Yongquan Huang, Xiaofei Zheng, Huiying Guo, Songwei Huan, Tao Jiang, Huajun Wang, Yulong Wei, Tao Gui

**Affiliations:** ^1^ Department of Bone and Joint Surgery The Affiliated Nanhua Hospital Hengyang Medical School University of South China Hengyang Hunan 421001 China; ^2^ Department of Orthopedics Medical Innovation Technology Transformation Center of Shenzhen Second People's Hospital The First Affiliated Hospital of Shenzhen University Shenzhen 518035 China; ^3^ Department of General Surgery The Second Affiliated Hospital of Bengbu Medical University Bengbu Anhui 233030 China; ^4^ Department of Sports Medicine The First Affiliated Hospital Guangdong Provincial Key Laboratory of Speed Capability The Guangzhou Key Laboratory of Precision Orthopedics and Regenerative Medicine Jinan University Guangzhou Guangdong 510630 China; ^5^ Department of Orthopaedics The First Hospital of China Medical University Shenyang 110001 China; ^6^ College of Chinese Materia Medical Tianjin University of Traditional Chinese Medicine Tianjin 301617 China; ^7^ State Key Laboratory of Traditional Chinese Medicine Syndrome/Department of Orthopaedics The Second Affiliated Hospital of Guangzhou University of Chinese Medicine Guangzhou Guangdong 510120 China; ^8^ Department of gerontology Guangzhou First People's Hospital Guangzhou Guangdong 510180 China; ^9^ Department of Bone and Joint Surgery The First Affiliated Hospital of Jinan University Guangzhou Guangdong 510632 China; ^10^ Department of Orthopaedics Union Hospital Tongji Medical College Huazhong University of Science and Technology Wuhan 430030 China

**Keywords:** CD74, CEBPB, luteolin, macrophage, osteoarthritis

## Abstract

Synovial inflammation represents a hallmark pathological process in osteoarthritis (OA), yet the cellular drivers orchestrating this response remain incompletely defined. Through single‐cell transcriptomic profiling of human OA synovial tissues, a distinct subset of CD74⁺ macrophages is identified that displayed robust pro‐inflammatory transcriptional signatures, underscoring the pivotal role of macrophages in shaping the inflammatory microenvironment. To explore therapeutic opportunities, computational ligand–target interaction analysis predicted luteolin as a high‐affinity binder of CD74. Mechanistically, luteolin suppressed CD74 expression and disrupted the assembly of the CEBPB–p65 complex, thereby preventing p65 nuclear translocation and subsequent activation of the NF‐κB signaling cascade in macrophages. To achieve targeted delivery, a nanoplatform MIF^79‐86^‐DS‐PLGA‐Luteolin (MDSPL) is engineered by conjugating MIF‐mimetic peptides onto reactive oxygen species (ROS)‐responsive Poly(lactic‐co‐glycolic acid) (PLGA) nanoparticles, enabling selective recognition of CD74⁺ macrophages. In vivo, MDSPL exhibited superior efficacy over free luteolin in attenuating synovial inflammation and halting OA progression. Notably, early intervention with MDSPL yielded stronger chondroprotective effects than delayed administration, highlighting the therapeutic value of timely targeting of macrophage‐driven inflammation. Collectively, these findings establish CD74⁺ macrophages as a pathogenic driver of OA‐associated synovial inflammation and introduce MDSPL as a precision nanotherapeutic strategy with translational potential for OA management.

## Graphical abstract

This study identifies CD74⁺ macrophages as key drivers of synovial inflammation in osteoarthritis (OA). The flavonoid luteolin is predicted to inhibit this pathway by blocking Nuclear Factor Kappa‐light‐chain‐enhancer of Activated B cells (NF‐κB) signaling. To enhance delivery, a targeted nanoplatform (MDSPL) is developed. MDSPL effectively reduced inflammation and halted OA progression in vivo, with early intervention proving most effective, offering a promising precision therapy for OA.

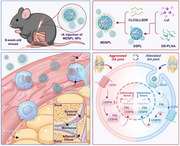

## 1 Introduction

Osteoarthritis (OA) represents a complex whole‐joint disorder characterized by progressive cartilage degradation, subchondral bone remodeling, and synovial inflammation.^[^ ^1^, ^2^ ^]^ While traditionally viewed primarily as a degenerative condition of articular cartilage, mounting evidence has established synovitis as a critical contributor to OA pathogenesis, disease progression, and symptom manifestation.^[^ ^3^ ^]^ The inflammatory changes within the synovial tissue not only exacerbate joint tissue destruction but also significantly influence patient outcomes through pain modulation and functional impairment.^[^ ^4^ ^]^ Among the various immune components within the inflamed synovium, macrophages have emerged as central regulators of the pathological process. Cartilage‐derived debris released into the joint cavity can be engulfed by macrophages, triggering their activation and subsequent secretion of pro‐inflammatory cytokines such as Interleukin‐1 beta (IL‐1β), tumor necrosis factor‐alpha (TNF‐α), and interleukin‐6 (IL‐6), along with cartilage matrix–degrading enzymes including matrix metalloproteinases (MMPs) and a disintegrin and metalloproteinase with thrombospondin motifs 4/5 (ADAMTS4/5),^[^ ^5^ ^]^ which in return cause more cartilage debris to be released into the joint cavity and further stimulate macrophages, thereby amplifying synovial inflammation and structural deterioration, forming a vicious cycle. Given their dual role as both initiators and amplifiers of the inflammatory cascade, synovial macrophages represent critical cellular mediators of OA pathogenesis, and strategies targeting this macrophage‐dependent vicious cycle hold considerable promise for therapeutic intervention.

CD74, also known as the invariant chain (Ii), is a type II transmembrane glycoprotein predominantly expressed on antigen‐presenting cells.^[^ ^6^ ^]^ Classically, CD74 serves as a chaperone for MHC class II molecules, but it also functions as the high‐affinity receptor for macrophage migration inhibitory factor (MIF). Upon MIF binding, CD74 undergoes regulated intramembrane proteolysis, releasing its intracellular domain (CD74‐ICD). The liberated CD74‐ICD translocates into the cytosol and nucleus, where it acts as a transcriptional regulator to initiate diverse downstream signaling cascades.^[^ ^6^, ^7^ ^]^ Beyond its canonical role in antigen presentation, CD74 has emerged as a pivotal mediator of immune responses and inflammatory processes, influencing cytokine regulation, immune cell activation, and disease pathogenesis.^[^ ^8^, ^9^ ^]^ For example, in non‐small cell lung cancer, human leukocyte antigen‐D related (HLA‐DR)⁺CD74⁺ neutrophils exhibit marked heterogeneity and plasticity, correlating with improved clinical outcomes and suggesting a role in anti‐tumor immunity.^[^ ^8^ ^]^ In neurodegenerative diseases such as Alzheimer's disease, CD74‐expressing microglia contribute to neuroinflammation by mediating responses to amyloid‐β–associated inflammatory stimuli.^[^ ^9^ ^]^ Within the tumor microenvironment, CD74‐expressing macrophages and dendritic cells interact with MIF to activate immunosuppressive pathways, thereby facilitating tumor immune evasion and dampening T cell responses.^[^ ^10^ ^]^ These findings underscore the diverse and context‐dependent roles of CD74 in shaping immune responses across pathological conditions. However, despite growing recognition of CD74 as a central regulator of inflammation and immunity, the existence and functional relevance of CD74‐expressing immune cell subsets in osteoarthritis (OA) remain poorly defined.

CCAAT/enhancer‐binding protein beta (CEBPB) is a transcription factor of the basic leucine zipper (bZIP) family that exerts critical regulatory functions in inflammation, immune cell differentiation, and cytokine production.^[^ ^11^ ^]^ CEBPB directly controls the transcription of key pro‐inflammatory mediators, including IL6, TNF‐α, and monocyte chemoattractant protein‐1 (MCP‐1), thereby shaping the inflammatory milieu. Dysregulated CEBPB activity has been widely implicated in chronic inflammatory disorders such as rheumatoid arthritis, atherosclerosis, and metabolic diseases, where its sustained activation amplifies pathological immune responses.^[^ ^12^, ^13^ ^]^ Importantly, CEBPB is indispensable for macrophage‐mediated inflammation, as it promotes the transition of macrophages toward a pro‐inflammatory phenotype, thereby perpetuating inflammatory cascades.^[^ ^14^ ^]^ These findings highlight CEBPB as a central node in immune regulation and a potential driver of chronic inflammation. Nevertheless, despite its established role in diverse inflammatory conditions, the specific contribution of CEBPB to OA pathogenesis remains poorly defined.

Natural small‐molecule compounds derived from traditional medicinal plants have demonstrated remarkable potential in modulating target protein expression and function due to their structural diversity, relatively low toxicity, and established safety profiles.^[^ ^15^ ^]^ However, the current paradigm in drug discovery predominantly relies on screening potential therapeutic molecules against existing disease models, which may overlook valuable therapeutic candidates. A significant challenge and research priority lies in developing efficient strategies for reverse screening of potential small‐molecule drugs based on identified protein targets. This approach would enable more targeted and efficient drug discovery processes, potentially accelerating the development of effective therapeutics. Luteolin (Lut), a naturally occurring flavonoid compound widely distributed across various plant species, has emerged as a particularly promising therapeutic agent. This bioactive molecule has garnered significant scientific interest due to its diverse biological activities and therapeutic potential ranging from inflammatory models to cancer treatment.^[^ ^15^, ^16^ ^]^ However, in most cases, the direct application of these small‐molecule compounds does not yield expected outcomes due to low bioavailability, poor solubility, insufficient safety, and lack of targeted distribution.^[^ ^17^ ^]^ Hence, nanoparticle (NP)‐based drug delivery systems have emerged as a revolutionary approach for addressing the issues. The ability to modify these nanocarriers' physicochemical properties allows for optimized drug loading, release kinetics, and cellular uptake, making them particularly attractive for inflammatory disease treatment. Among various nanocarrier systems, poly (lactic‐co‐glycolic acid) (PLGA) has garnered substantial attention in the biomedical field due to its outstanding biocompatibility, controlled biodegradability, and well‐established safety profile, as evidenced by its food and drug administration (FDA) approval for various biomedical applications.^[^ ^18^ ^]^ However, conventional PLGA NPs face a critical limitation in their non‐specific distribution pattern within synovial tissue. The widespread uptake of these NPs by various cell types in the synovial membrane compromises their ability to achieve targeted delivery to specific cell subpopulations.^[^ ^19^ ^]^ This non‐selective distribution pattern not only reduces therapeutic efficiency but also increases the potential for unintended effects in non‐target cells. The challenge of developing delivery systems that can specifically target distinct macrophage subsets within the complex synovial microenvironment has become increasingly important.

In this study, we performed integrated single‐cell RNA sequencing (scRNA‐seq) analysis of human synovial tissues and identified a distinct macrophage subpopulation defined by elevated CD74 expression (CD74⁺ macrophages). Cross‐species validation through histological and cellular analyses confirmed that this subset exhibits pronounced pro‐inflammatory properties, highlighting its potential role in OA–associated synovial inflammation. To explore therapeutic opportunities, we applied computational drug screening and identified luteolin as a candidate CD74‐binding compound. Mechanistic investigations revealed that Luteolin attenuates macrophage inflammatory responses by disrupting a CD74‐dependent signaling axis, specifically impairing CEBPB–p65 complex formation, preventing p65 nuclear translocation, and ultimately suppressing NF‐κB pathway activation. Building upon this mechanistic insight, we designed a precision nanotherapeutic system to selectively target CD74⁺ macrophages. Specifically, we engineered reactive oxygen species (ROS)‐responsive PLGA nanoparticles cross‐linked via diselenide bonds (PLGA‐Se‐Se‐PEG‐MAL, DS‐PLGA) and functionalized them with a MIF‐mimetic peptide (LCGLLSDR, residues 79–86 of MIF), thereby enabling specific recognition of CD74. This nanoplatform, termed **MDSPL**, facilitated targeted luteolin delivery and achieved selective inhibition of pro‐inflammatory CD74⁺ macrophages. Collectively, our findings identify CD74⁺ macrophages as key contributors to OA pathogenesis and introduce MDSPL as an innovative nanotherapy with the potential to provide more effective and selective treatment of synovial inflammation.

## 2 Results

### 2.1 CD74⁺ Macrophages Represent a Pro‐Inflammatory Subset in Osteoarthritic Synovial Tissues

**Figure 1:**
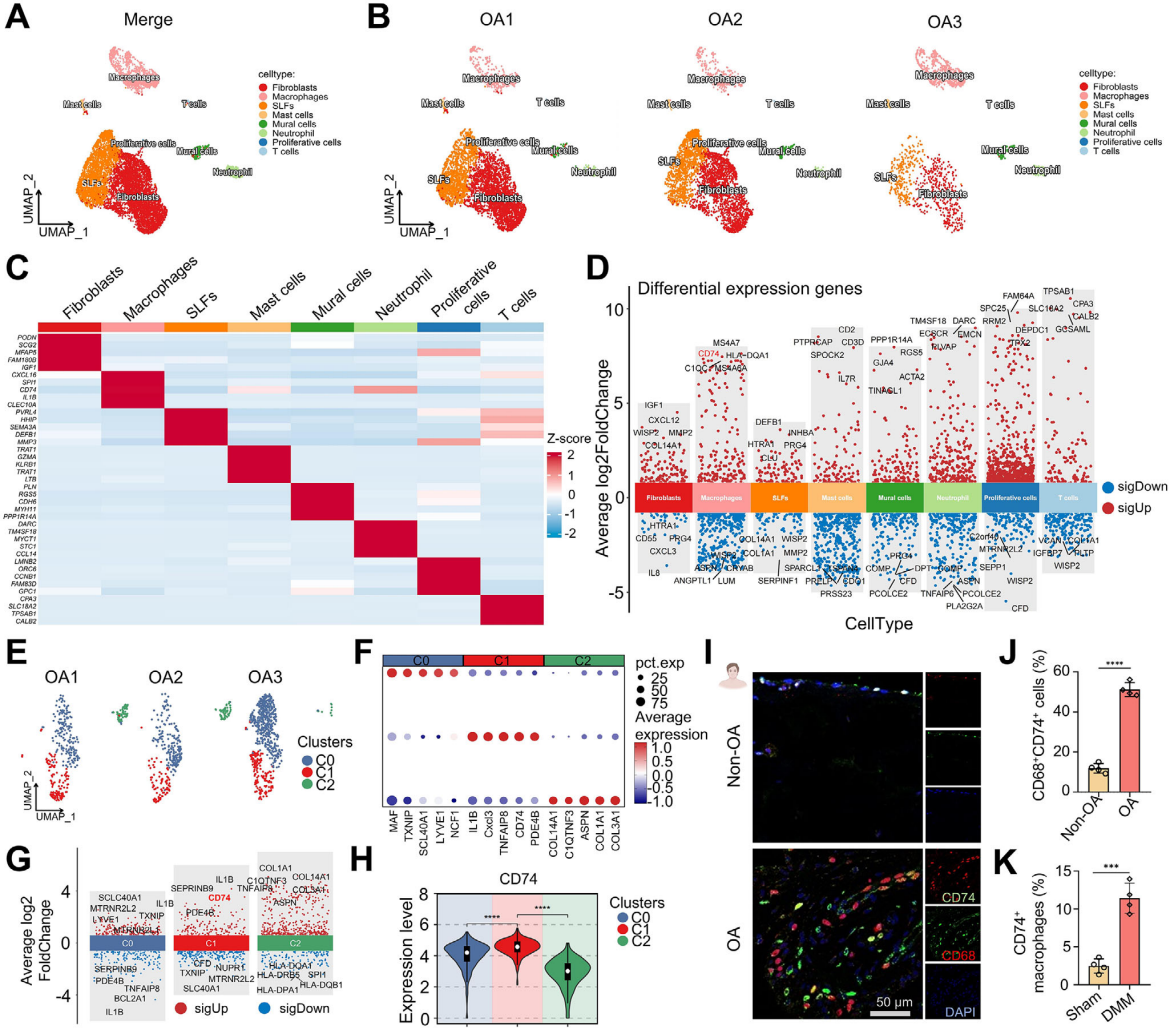
Discovery of a novel CD74⁺ macrophage subset in synovial tissues of OA. A,B) The UMAP dimensional reduction analysis of scRNA data from synovial tissues in clinical patients with late‐stage OA. C) The heatmap analysis displaying the expression patterns of canonical cell‐type‐specific marker genes across eight identified synovial cell subpopulations of human tissues. D) The volcano map of DEGs across eight identified human synovial cell subpopulations. E) The UMAP visualization of macrophage subpopulations from human synovial tissue scRNA datasets. F) Comprehensive dot plot visualization displaying the expression patterns of marker genes across Cluster C0, C1, and C2 macrophage cell subpopulations. G) The volcano map of DEGs of three macrophage subpopulations. H) Detailed violin plot analysis showing the relative expression levels of CD74 across the Cluster C0, C1, and C2 macrophage cell subpopulations in human synovial tissues. I,J) Representative IF images and quantification of CD74^+^CD68^+^ cells in synovial tissues of the Non‐OA and OA group. Scale bar: 50 µm. K) The percentage of CD74^+^ macrophages in the mouse OA synovial tissues. (*n* = 4 independent biological replicates per group). The data for all bar graphs is presented as mean ± SD, and P values were calculated by two‐tailed unpaired Student's t‐test. ****P < 0.0001.

To investigate the cellular heterogeneity of synovial tissues during OA progression, we first analyzed scRNA‐seq data obtained from synovial tissues of three OA patients undergoing total knee arthroplasty. Unsupervised clustering based on canonical marker gene expression identified eight transcriptionally distinct cell populations, including fibroblasts, macrophages, synovial lining fibroblasts (SLFs), mast cells, mural cells, neutrophils, proliferative cells, and T cells (**Figure** **1**A–C). To our interest, differential gene expression analysis across these clusters revealed that CD74 was one of the most significantly upregulated genes within the macrophage population (Figure 1D), highlighting its potential functional relevance in OA‐associated synovial inflammation. Further analysis revealed that macrophages accounted for ≈85.99% of CD74‐expressing cells, whereas other immune populations, including T and B cells, contributed less than 15% (Figure S1A, Supporting Information). The CD74 ligand MIF was predominantly expressed by fibroblasts (Figure S1B, Supporting Information), with PDGFRα⁺ fibroblasts showing markedly elevated MIF levels in early OA compared with sham controls (Figure S1C, Supporting Information), suggesting a potential role of MIF–CD74 signaling axis in mediating synovial inflammation during osteoarthritis progression. To further delineate the heterogeneity within synovial macrophages, we performed subclustering and identified three discrete subsets, designated C0, C1, and C2 (Figure 1E,F). Strikingly, the C1 subset exhibited marked enrichment of CD74 expression compared with the other subsets (Figure 1G,H). Based on this observation, this population was classified as CD74⁺ macrophages. Co‐localization studies further demonstrated elevated accumulation of CD74 together with the macrophage marker CD68 in clinical OA synovial tissues, confirming the increased prevalence of CD74⁺ macrophages under osteoarthritic conditions (Figure 1I,J). To corroborate these findings in an in vivo model, we conducted flow cytometry analysis of synovial macrophages isolated from mouse Sham and OA groups. Macrophages were defined as CD45⁺CD11b⁺F4/80⁺ and further subdivided into CD74^−^ and CD74⁺ fractions (Figure S1D,E, Supporting Information). OA synovial tissues displayed a substantial increase in the proportion of CD74⁺ macrophages compared with Sham controls (Figure 1K), demonstrating that the expansion of this subset is a conserved feature of OA pathology across species.

**Figure 2:**
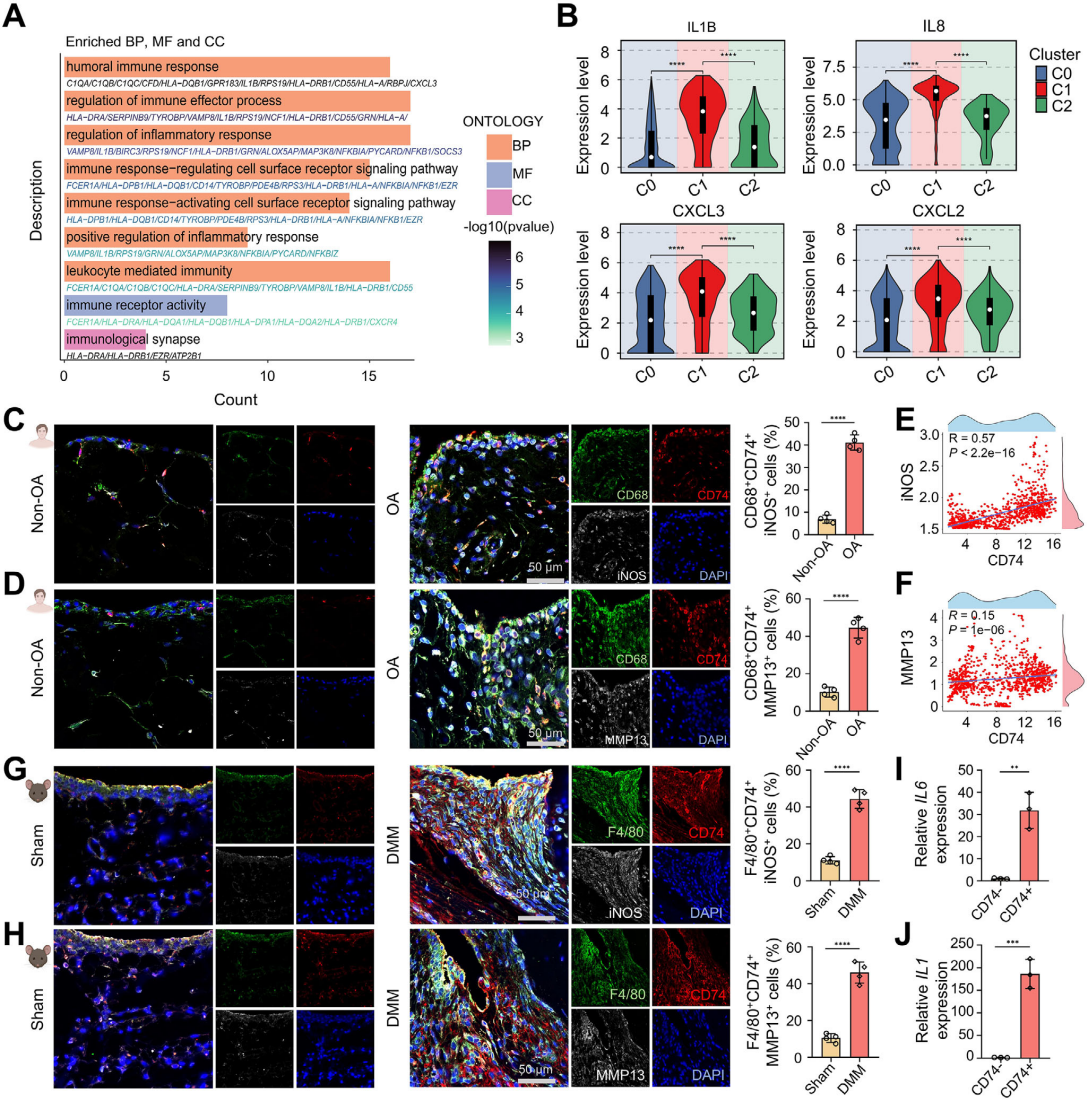
CD74⁺ macrophages represent a pro‐inflammatory subset in osteoarthritic synovial tissues. A) The GO enrichment analysis of C1‐macrophage subpopulations (CD74^high^ macrophages) in human synovial tissues. B) Detailed violin plot analysis showing the relative expression levels of IL1B, IL8, CXCL2, and CXCL3 across the C0, C1, and C2 macrophage subpopulations in human synovial tissues. C,D) Spatial analysis of CD74‐expressing macrophages in synovial tissues. Left: Representative IF images showing co‐expression of CD74 (red) and iNOS (white) or MMP13 (white) with the human macrophage marker CD68 (green). Nuclei are visualized with DAPI (blue). Right: Quantitative assessment of CD68^+^CD74^+^iNOS^+^ and CD68^+^CD74^+^MMP13^+^ cells (*n* = 4 independent biological replicates per group). Scale bar: 50 µm. E) Pearson correlation analysis between CD74 and iNOS in CD74^high^ macrophages from synovial tissues of human samples. F) Pearson correlation analysis between CD74 and MMP13 in CD74^high^ macrophages from synovial tissues of human samples. G,H) Spatial analysis of CD74‐expressing macrophages in synovial tissues. Left: Representative IF images showing co‐expression of CD74 (red) and iNOS (white) or MMP13 (white) with the mouse macrophage marker F4/80 (green). Nuclei are visualized with DAPI (blue); Right: Quantitative assessment of F4/80^+^CD74^+^iNOS^+^ and F4/80^+^CD74^+^MMP13^+^ triple‐positive cells. Scale bar: 50 µm. I,J) Quantitive analysis of *IL6* and *IL1* mRNA expression levels in CD74‐N and CD74‐P macrophage populations sorted by FACS (each data point represents a measurement from a pool of tissues collected from 8 mice subjected to DMM surgery, with a total of 3 biological replicates conducted for comparison). The data for all bar graphs is presented as mean ± SD, and P values were calculated by (C, D, G, H and I‐J) two‐tailed unpaired Student's t‐test. **P < 0.01, ***P < 0.001, ****P < 0.0001.

We next sought to investigate the functional properties of CD74⁺ macrophages. Gene ontology (GO) enrichment analysis of this subset revealed significant association with pathways related to inflammation and immunity (**Figure** **2**A). Transcriptional comparisons across the three macrophage subsets demonstrated that CD74⁺ macrophages (C1) expressed markedly higher levels of pro‐inflammatory mediators such as IL1B, IL8, CXCL3, and CXCL2 compared with C0 and C2 subsets (Figure 2B). To experimentally validate the pro‐inflammatory phenotype of CD74⁺ macrophages, we examined the spatial expression of CD74 in relation to the macrophage marker CD68 and two well‐established inflammatory mediators in clinical synovial tissues, iNOS and MMP13. Compared with Non‐OA controls, OA synovial tissues exhibited pronounced co‐expression of CD74 with CD68, iNOS, and MMP13 (Figure 2C,D). Quantitative fluorescence‐based correlation analysis revealed significant positive correlations between CD74 protein levels and those of iNOS and MMP13 (Figure 2E,F). These findings provide strong evidence that CD74⁺ macrophages represent an inflammation‐associated subset in OA synovium. Importantly, these results were recapitulated in murine OA tissues, where enhanced co‐localization of CD74 with F4/80, iNOS and MMP13 was also observed compared with Sham tissues (Figure 2G,H). To further establish the functional characteristics of this macrophage subset, we sorted CD74⁺ and CD74^−^ primary macrophages from murine OA synovial tissues (Figure S1D,E, Supporting Information). Transcriptomic profiling revealed that CD74⁺ macrophages expressed substantially higher levels of key pro‐inflammatory cytokines, including *IL1* and *IL6*, compared with CD74^−^ macrophages (Figure 2I,J). Taken together, our findings identify CD74⁺ macrophages as a unique subset enriched in both human and murine OA synovial tissues. The consistent presence of CD74⁺ macrophages across species and their strong correlation with the inflammatory microenvironment suggest that they represent a conserved cellular mechanism contributing to OA‐associated synovial inflammation. These results highlight CD74⁺ macrophages as potential drivers of OA progression and underscore their relevance as a promising therapeutic target in the management of OA.

### 2.2 Luteolin Acts as a Promising Small‐Molecule Agent in Mediating the CD74‐Related Macrophage Inflammation Process

**Figure 3:**
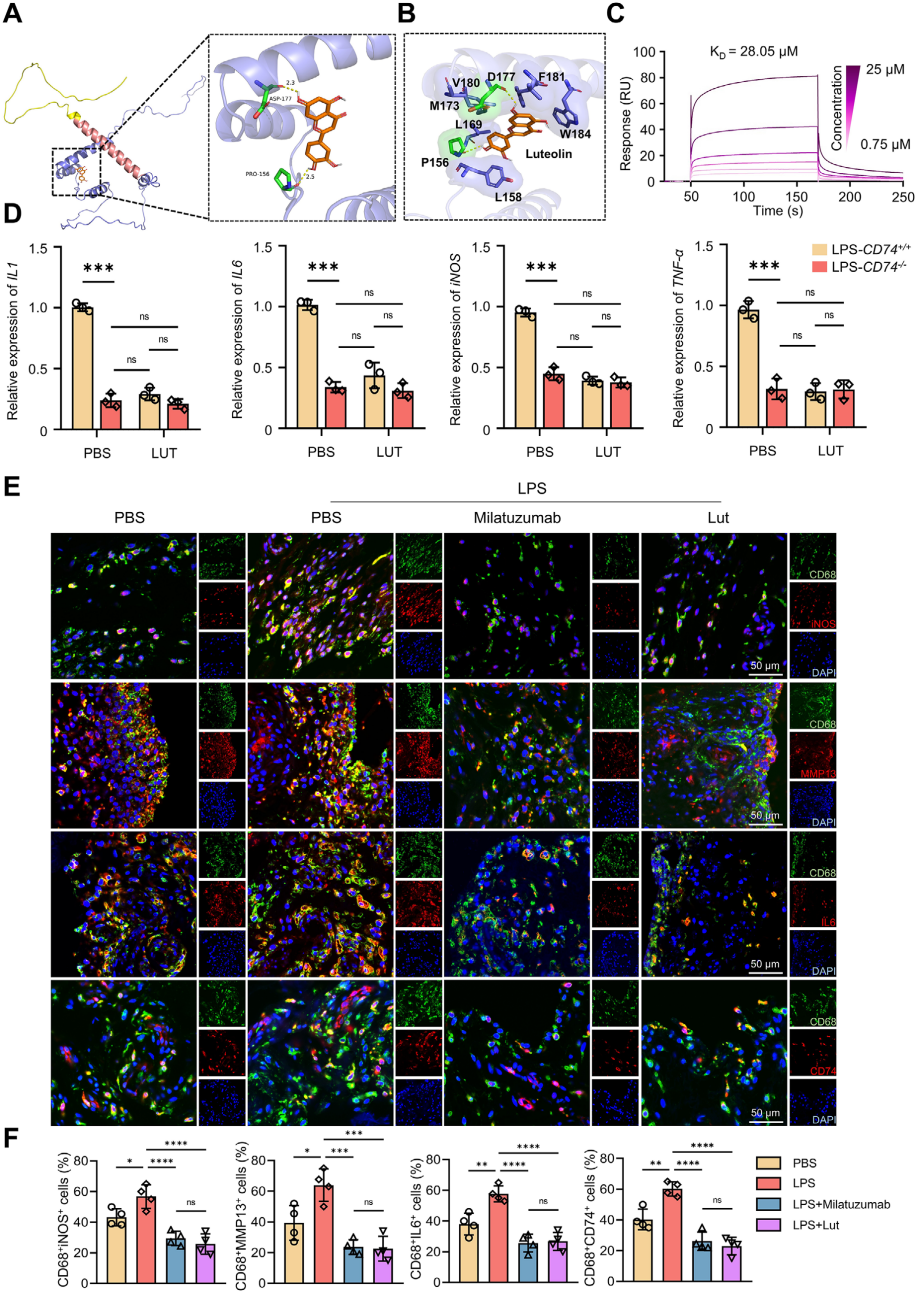
Luteolin acts as a promising small molecule agent in mediating CD74‐related macrophage inflammation process. A) Visualization of the docking results between CD74 and Lut compound. B) The 2D binding modes showcasing the interaction pattern of Lut with binding pocket residues of CD74 protein. C) Real‐time analysis of the direct interaction between CD74 and luteolin by Surface Plasmon Resonance (SPR). D) Transcriptional levels of *IL‐1β*, *IL‐6*, *iNOS*, and *TNF‐α* in LPS‐stimulated RAW 264.7 cells with or without CD74 knockout, treated with PBS or Lut. (*n* = 3 independent biological replicates per group). E) Characterization of iNOS‐, MMP13‐, IL6‐, and CD74‐ expressing CD68^+^ cells in human synovial explants. Representative IF images showing co‐localization of CD68 (green) with the iNOS (red), MMP13 (red), IL6 (red) or CD74 (red). Nuclei are visualized with DAPI (blue). F) Quantitative assessment of CD68^+^iNOS^+^, CD68^+^MMP13^+^ cells, CD68^+^IL6^+^ cells and CD68^+^CD74^+^ cells (*n* = 3 independent biological replicates per group). The data are presented as mean ± SD. P values were calculated by (D and G‐J) one‐way ANOVA. *P < 0.05, **P < 0.01, ***P < 0.001, ****P < 0.0001. ns: not significant.

Given the fact that CD74^+^ macrophages were inflammatory cells both in human and murine OA synovial tissues (Figures 1 and 2), which makes it a promising target for modulating OA‐related inflammatory response. To achieve this, prioritized screening for anti‐inflammatory agents that distinctly bind to CD74 was carried out. Due to the absence of a reported crystal structure for CD74, First, homology modeling was performed to successfully obtain a predicted CD74 structure consisting of the intracellular region, transmembrane region, and extracellular segment (Figure S2A–D, Supporting Information). Then, five target pockets with their properties, including volume, surface area, and depth of CD74, were identified and listed in Table S1, from which pocket_1 was selected as the protein pocket for virtual screening (Figure S2E, Supporting Information). Through virtual screening, we identified the top 25 potential molecules (Table S2, Supporting Information) and visualized the docking results of the top five molecules with the CD74 protein, including Luteolin (Lut, −10.4 kcal mol^−1^), Albanol (−9.6 kcal mol^−1^), Lnophyllum E (−9.5 kcal mol^−1^), Oxysanguinarine (−7.9 kcal mol^−1^), and β‐carotene (−7.9 kcal mol^−1^). Among these, Lut exhibited the strongest binding affinity to the intracellular domain of CD74 (**Figure** **3**A,B; Figure S2F, Supporting Information), making it a promising candidate for further investigation in CD74‐mediated macrophage inflammatory response in OA.

To determine the optimal dosage for Lut, dose‐dependent administration of Lut was conducted on RAW 264.7 cells stimulated with lipopolysaccharide (LPS), with results revealing that a concentration of 30 µg mL^−1^ of Lut exhibited the most significant anti‐inflammatory effects without compromising cell viability (Figure S3A,B, Supporting Information). At the protein level, Lut treatment significantly suppressed LPS‐induced CD74 expression in RAW 264.7 cells (Figure S3C, Supporting Information), which further verified molecular docking results. In addition, synovial inflammatory proteins, including MMP13 and iNOS, were also markedly decreased upon Lut treatment (Figure S3C, Supporting Information). Additionally, Lut effectively attenuated the LPS‐induced production of reactive oxygen species (ROS) in macrophages (Figure S3D, Supporting Information). Given the pronounced anti‐inflammatory effects of Lut on macrophages and its ability to downregulate CD74 expression, we next sought to determine whether Lut's anti‐inflammation activity is mediated through direct interaction with CD74. To address this, we performed surface plasmon resonance (SPR) analysis to quantitatively assess Lut binding to the intracellular domain of CD74, which was immobilized on the SPR sensor chip in accordance with our molecular docking studies. Real‐time sensorgrams revealed a clear dose‐dependent binding response, yielding an equilibrium dissociation constant (K_D_) of 28.05 µm (Figure 3C), consistent with the affinity predicted by molecular docking. These biophysical results provide strong evidence for a specific Lut–CD74 interaction. Functionally, CD74 knockdown (*CD74^−/−^*, Figure S4A–D, Supporting Information) in BMDMs abrogated the ability of Lut to suppress inflammatory gene expression (Figure 3D), indicating that CD74 is required for Lut's anti‐inflammatory activity. To further explore the mechanism, we assessed whether Lut alters CD74 protein stability using cycloheximide (CHX) chase assays. CHX, a translational inhibitor that prevents new protein synthesis,^[^ ^20^ ^]^ was applied to LPS‐stimulated macrophages with or without Lut treatment, and degradation kinetics were monitored. In the absence of Lut, CD74 protein was degraded within 12 h. However, when macrophages were treated with both CHX and Lut, the degradation of CD74 was significantly accelerated, with a much faster reduction in protein levels (Figure S4E,F, Supporting Information). To further validate the anti‐inflammatory potential of Lut in a more clinically relevant setting, an ex vivo model using synovial tissue explants from late‐stage OA patients after total knee joint arthroplasty surgeries was employed (Figure S4G, Supporting Information). Given the structural integrity constraints of intact explants, CD74 knockdown was not technically feasible in this setting. Therefore, we adopted a humanized anti‐CD74 monoclonal antibody (Milatuzumab) as a pharmacological positive control to inhibit CD74 signaling. We implemented the following groups: PBS, LPS + PBS, LPS + Milatuzumab, and LPS + Lut. Immunofluorescence and quantitative analyses of synovial explants demonstrated that Lut exerted anti‐inflammatory effects comparable to Milatuzumab, with no statistically significant differences between Lut and Milatuzumab across the measured inflammatory factors (Figure 3E,F). These data support that CD74 inhibition recapitulates the anti‐inflammatory phenotype observed with Lut. Taken together, our data indicate that Lut exerts anti‐inflammatory effects by directly binding to CD74 in synovial macrophages and inhibiting their inflammatory phenotype, which likely contributes to the attenuation of OA‐associated synovitis and cartilage degradation. However, we do not exclude the possibility that Lut may also act on other cell types, such as chondrocytes, to exert protective effects.

### 2.3 Luteolin Inhibits Macrophage Inflammatory Response through the CD74/CEBPB/P65 Axis

**Figure 4:**
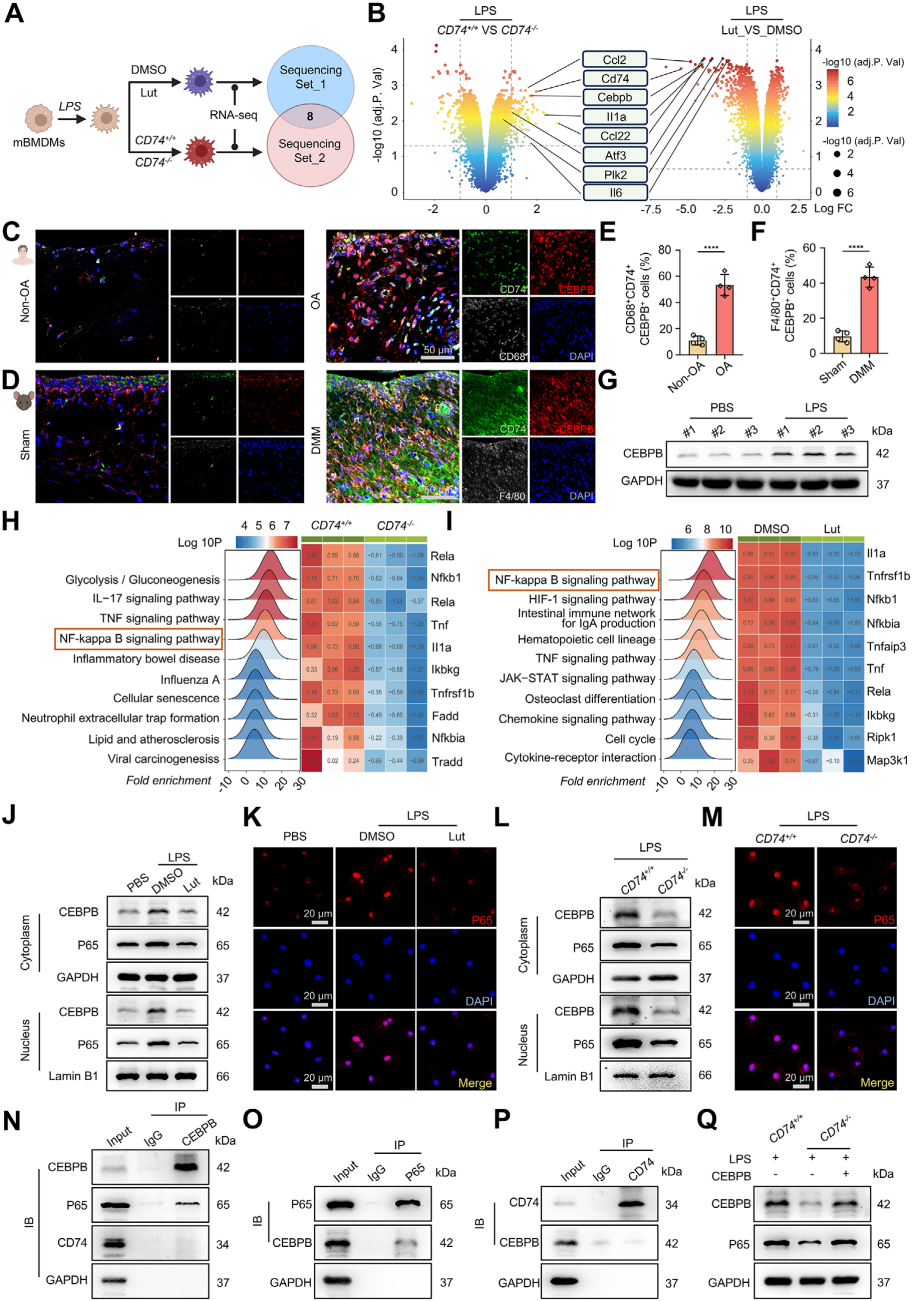
Luteolin inhibits macrophage inflammatory response through the CD74/CEBPB/P65 axis. A) Schematic diagram of screening the common targets in macrophages regulated by both Lut and CD74 via two independent RNA‐seq experiments. B) The volcano map showing the common down‐regulated DEGs obtained from two independent RNA‐seq results. C) Representative fluorescence images showing CD74 (green), CEBPB (red) and CD68 (white) co‐localization in synovial tissues from the Non‐OA and OA group. Scale bar: 50 µm. D) Representative fluorescence images showing CD74 (green), CEBPB (red) and F4/80 (white) co‐localization in synovial tissues from the Sham and early DMM group (7 days after the DMM modeling surgery). Scale bar: 50 µm. E) Quantitative assessment of the percentage of CD68^+^CD74^+^CEBPB^+^ triple‐positive cells in synovial tissues from the Non‐OA and OA group (n = 4 independent biological replicates per group). F) Quantitative assessment of the percentage of F4/80^+^CD74^+^CEBPB^+^ triple‐positive cells in synovial tissues from the Sham and DMM group (n = 4 independent biological replicates per group). G) IB analysis detecting the protein amount of CEBPB in RAW 264.7 cells treated with PBS or LPS. H) The KEGG enrichment analysis comparing macrophages from the *CD74^+/+^* and *CD74^−/−^* group. Left panel: The peak map shows the enrichment degree of the signaling pathway; Right panel: The heat map shows the difference in gene expression from NF‐κB signaling pathway between *CD74^+/+^* and *CD74^−/−^* group. I) The KEGG enrichment analysis comparing macrophages from DMSO‐ and Lut‐group. Left panel: The peak map shows the enrichment degree of the signaling pathway; Right panel: The heat map shows the difference in gene expression from NF‐κB signaling pathway between DMSO‐ and Lut‐treated group. J) IB analysis detecting cytoplasmic and nuclear protein amounts of CEBPB and P65 in primary mBMDMs activated by LPS with or without Lut treatment. K) Representative fluorescence images showing p65 localization in primary mBMDMs activated by LPS with or without Lut treatment. Scale bar: 20 µm. L) IB analysis detecting cytoplasmic and nuclear protein amounts of CEBPB and P65 in *CD74^+/+^* and *CD74^−/−^* primary mBMDMs under LPS activation. M) Representative fluorescence images showing p65 localization in *CD74^+/+^* and *CD74^−/−^* primary mBMDMs under LPS activation. Scale bar: 20 µm. N) Co‐IP assay detecting the endogenous interaction of CEBPB with P65 and CD74, respectively. O) Co‐IP assay detecting the endogenous interaction of P65 with CEBPB. P) Co‐IP assay detecting the endogenous interaction of CD74 with CEBPB. Q) IB analysis detecting the protein amounts of CEBPB and P65 in *CD74^+/+^‐* and *CD74^−/−^‐*derived primary mBMDMs transfected with or without CEBPB‐overexpression plasmids in the background of LPS activation. The data are presented as mean ± SD. P values were calculated by (E‐F) two‐tailed unpaired Student's t‐test. ****P < 0.0001.

To elucidate the molecular mechanisms underlying Lut‐mediated regulation of CD74‐associated macrophage inflammation, we performed two parallel high‐throughput transcriptome sequencing experiments (**Figure** **4**A). The first dataset (Sequencing Set_1) compared LPS‐stimulated macrophages with or without Lut treatment, while the second (Sequencing Set_2) compared LPS‐stimulated bone marrow–derived macrophages (mBMDMs) from *CD74⁺/⁺* and *CD74^−^/^−^* mice (Figure S4A–D, Supporting Information). This dual approach enabled the identification of shared regulatory pathways affected by both Lut intervention and genetic ablation of CD74. Comparative analysis of differentially expressed genes (DEGs) across the two datasets revealed eight genes commonly downregulated by both Lut treatment and CD74 deficiency (Figure 4B). Among these, Cebpb, activating transcription factor 3 (Atf3), and polo like kinase 2 (Plk2) were prioritized as candidate targets for further investigation. Subsequent validation confirmed that Lut significantly suppressed *CEBPB* expression, whereas *ATF3* and *PLK2* exhibited inconsistent responses (Figure S5A, Supporting Information), implicating CEBPB as a downstream effector of CD74‐dependent inflammatory signaling. Supporting this, enhanced co‐localization of CEBPB with CD74 and macrophage markers (CD68 in human tissues; F4/80 in murine tissues), together with a positive correlation between CD74 and CEBPB protein levels, suggested coordinated regulation under OA inflammatory conditions (Figure 4C–F; Figure S5B, Supporting Information). Further analysis in RAW 264.7 cells demonstrated that LPS stimulation markedly increased CEBPB protein expression (Figure 4G). These findings reveal that CEBPB is spatially and functionally associated with CD74⁺ macrophages and acts as a pro‐inflammatory mediator.

The NF‐κB pathway, with P65 at its core, is a critical node in the cellular response to inflammation.^[^ ^21^ ^]^ Upon activation, P65 translocates into the nucleus, initiating a transcriptional program that promotes inflammatory gene expression.^[^ ^22^ ^]^ KEGG enrichment analysis revealed significant activation of the NF‐κB pathway both in CD74‐deficient macrophages and in Lut‐treated cells (Figure 4H,I), and this enrichment was further confirmed in human CD74⁺ macrophages (Figure S5C, Supporting Information). These transcriptomic findings prompted the hypothesis that Lut suppresses macrophage inflammation through a CD74–CEBPB–NF‐κB axis. To test this, immunoblotting was employed to assess whether Lut modulates NF‐κB activation via inhibition of P65 nuclear translocation. Lut treatment markedly reduced both cytoplasmic and nuclear levels of P65 and CEBPB (Figure 4J), accompanied by a pronounced decrease in P65 nuclear translocation in primary mBMDMs (Figure 4K; Figure S6A, Supporting Information). Parallel experiments in *CD74^−^/^−^* mBMDMs demonstrated similar reductions in CEBPB and P65 expression in both compartments (Figure 4L), as well as diminished nuclear translocation of P65 compared with *CD74⁺/⁺* controls (Figure 4M; Figure S6B, Supporting Information), supporting a CD74‐dependent regulation of NF‐κB signaling. In line with this, in LPS‐activated RAW 264.7 cells, both Lut treatment and CD74 knockdown by small interfering RNA (siRNA) produced concordant effects, significantly impairing P65 nuclear translocation (Figure S6C–F and Table S3, Supporting Information). These findings provide robust evidence that Lut attenuates NF‐κB pathway activation by disrupting nuclear translocation of P65 in a CD74‐dependent manner.

Given that both cytoplasmic and nuclear levels of CEBPB and P65 were reduced following Lut treatment or CD74 knockout, we next investigated whether these proteins physically interact in the context of CD74‐mediated macrophage inflammation. Co‐immunoprecipitation (Co‐IP) assays revealed a robust interaction between CEBPB and P65 (Figure 4N,O), suggesting their cooperative involvement in regulating inflammatory responses. By contrast, no direct protein–protein interaction was detected between CD74 and CEBPB (Figure 4N,P), indicating that CD74 influences CEBPB activity indirectly. To further validate that CD74 exerts its effects on NF‐κB signaling via CEBPB, macrophages were transfected with a CEBPB overexpression plasmid prior to LPS stimulation (Figure S7A–C and Table S4, Supporting Information). Overexpression of CEBPB markedly diminished the anti‐inflammatory effects observed in CD74‐deficient macrophages, as evidenced by elevated inflammatory marker expression, increased P65 levels, and enhanced nuclear translocation of P65 (Figure 4Q; Figure S7D,E, Supporting Information), suggesting that CEBPB functions as a critical mediator linking CD74 to NF‐κB activation. Collectively, these findings provide evidence that the anti‐inflammatory effects of Lut on macrophages are mediated through the inhibition of CD74, which subsequently interrupts the interaction between CEBPB and P65 and the activation of the NF‐κB pathway.

### 2.4 Luteolin Attenuates Inflammatory Crosstalk between Macrophages and Chondrocytes and Demonstrates Therapeutic Efficacy In Vivo

**Figure 5:**
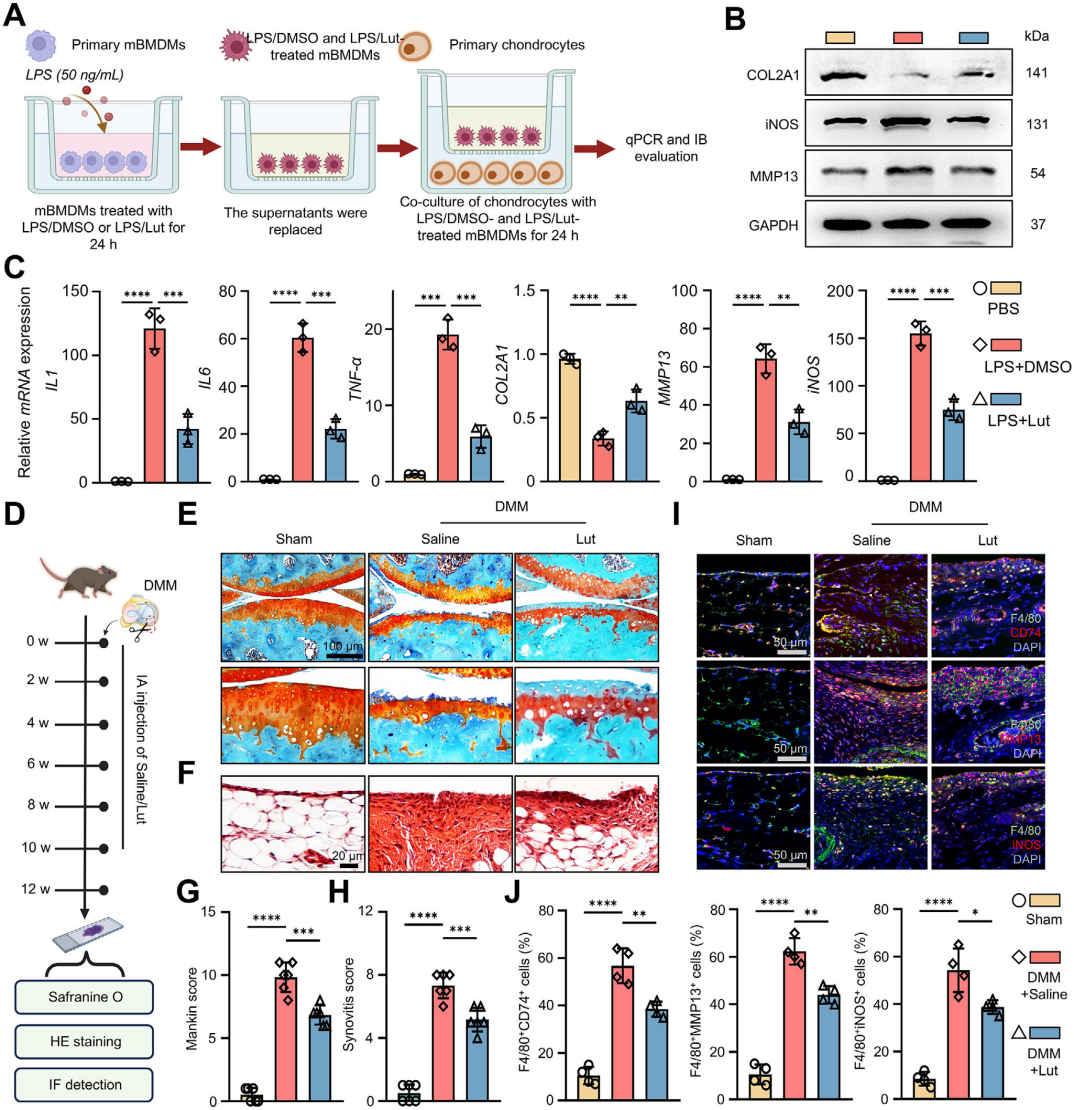
Luteolin attenuates inflammatory crosstalk between macrophages and chondrocytes and shows certain therapeutic effects in vivo. A) A comprehensive experimental protocol to evaluate the therapeutic efficacy of Lut in attenuating inflammatory crosstalk between macrophages and chondrocytes in vitro. B) IB analysis detecting the protein amounts of COL2A1, iNOS and MMP13 in chondrocytes co‐cultured with macrophages treated with LPS with or without Lut. C) Transcription levels of *IL1*, *IL6*, *TNF‐α* in macrophages, and *COL2A1*, *MMP13*, and *iNOS* in chondrocytes co‐cultured with LPS‐ and LPS/Lut‐treated mBMDMs (*n* = 3 independent biological replicates per group). D) A comprehensive experimental protocol to evaluate the therapeutic efficacy of Lut in DMM‐induced OA mouse model, with detailed intervention timelines and treatment schedules as illustrated in the experimental scheme. E,F) Representative Safranin O/Fast green (Scale bar: 50 µm) and HE staining images (Scale bar: 20 µm) of knee joints at 12 weeks after DMM surgery. G,H) Quantitative assessment of OA severity with Mankin score and synovial score (*n* = 6 independent biological replicates per group). I) Representative fluorescence images in synovial tissues of the Sham and DMM group (12 weeks after the DMM modeling surgery), showing CD74, MMP13, and iNOS co‐localization in macrophages (co‐localized with F4/80). Scale bar: 50 µm. J) Quantitative assessment showing CD74, MMP13 and iNOS co‐localization percentage with F4/80 in synovial tissues from the mouse treated with different treatments (*n* = 4 independent biological replicates per group). The data are presented as mean ± SD. The data are presented as mean ± SD. P values were calculated by (C and G‐J) one‐way ANOVA. *P < 0.05, **P < 0.01, ***P < 0.001, ****P < 0.0001.

In OA, synovial inflammation and cartilage degradation are tightly interconnected, forming a self‐perpetuating cycle in which pro‐inflammatory cytokines sustain inflammation and accelerate cartilage erosion.^[^ ^23^, ^24^ ^]^ To mimic this pathological microenvironment and evaluate the chondroprotective effects of Lut, a co‐culture system of primary chondrocytes and LPS‐stimulated mBMDMs was established, with macrophages treated either with Lut or dimethyl sulfoxide (DMSO) (**Figure** **5**A). Chondrocytes co‐cultured with DMSO‐treated macrophages exhibited pronounced upregulation of iNOS and MMP13, together with significant downregulation of the cartilage matrix protein collagen type II alpha 1 chain (COL2A1). By contrast, co‐culture with Lut‐treated macrophages markedly reduced iNOS and MMP13 expression while restoring COL2A1 levels (Figure 5B,C). Consistent with these observations, analysis of macrophages in the co‐culture revealed that Lut treatment suppressed the inflammatory secretome, including *IL1*, *IL6*, and *TNF‐α* (Figure 5C), underscoring the ability of Lut to protect chondrocytes by mitigating macrophage‐mediated inflammatory signaling.

To further assess its therapeutic relevance, Lut was evaluated in a destabilization of the medial meniscus (DMM)‐induced murine OA model, with intra‐articular injections of Lut or saline following surgery (Figure 5D). 12 weeks after DMM, saline‐treated mice exhibited severe cartilage erosion, loss of superficial and intermediate layers, accumulation of hypertrophic chondrocytes, and elevated Mankin and synovitis scores, accompanied by synovial thickening and immune cell infiltration. In contrast, Lut‐treated mice maintained relatively preserved tibial cartilage, with reduced fibrillation and clefts, and demonstrated significantly lower Mankin and synovitis scores (Figure 5E–H). Consistent with this, Lut treatment reduced the levels of CD74, MMP13, and iNOS in macrophages within the synovium (Figure 5I,J). These findings suggest that Lut inhibits the inflammatory phenotype of synovial macrophages, potentially contributing to the attenuation of synovial inflammation, preservation of cartilage integrity.

### 2.5 MIF‐CD74 Interaction Grounds the Construction of a Nano‐Based Luteolin Delivery System

**Figure 6:**
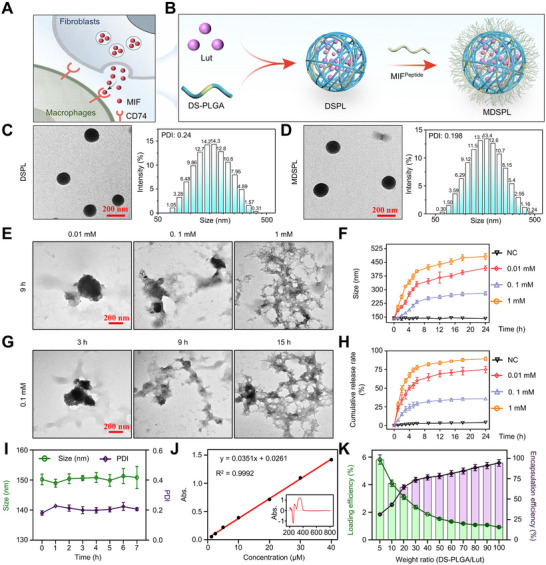
MIF‐CD74 interaction grounds the construction of nano‐based luteolin delivery system. A) Schematic representation illustrating the molecular interaction between fibroblast‐secreted MIF and CD74 on macrophage, highlighting key interaction interfaces crucial for targeted drug delivery. B) Schematic representation illustrating the design rationale and formulation process of Lut‐loaded PLGA NPs decorated with polypeptide that mimics MIF protein in OA treatment. C,D) Left panel: TEM image of DSPL and MDSPL showcasing the morphology and size distribution of the particles. Scale bar: 200 nm. Right panel: DLS analysis of DSPL and MDSPL dispersion, illustrating the hydrodynamic diameter distribution. E,F) TEM images illustrating the ROS‐responsive disintegration of MDSPL. The images show a progressive increase in particle size and disintegration rate with rising concentrations of hydrogen peroxide_._ G) TEM images illustrating the time‐dependent ROS‐responsive disintegration of MDSPL with pointed concentrations of hydrogen peroxide_._ H) Cumulative release rate curves of the MDSPL over time, demonstrating the percentage of Lut released from the MDSPL by pointed time (*n* = 3 independent biological replicates per group). I) The stability of MDSPL at different times in PBS (*n* = 3 independent biological replicates per group). J) Concentration‐UV absorbance curves of the Lut illustrate the relationship between UV absorbance and different drug concentrations, with the UV absorption spectrum of the Lut inserting in the inner part. K) The drug loading capacity and encapsulation efficiency of DS‐PLGA with different weight ratios of Lut (*n* = 3 independent biological replicates per group). The data are presented as mean ± SD.

Although Lut has demonstrated potent anti‐inflammatory properties in vitro, its therapeutic efficacy in vivo is hindered by poor bioavailability, rapid clearance, and limited targeted delivery to the desired tissues.^[^ ^25^ ^]^ To overcome these challenges and enhance the targeted delivery of Lut to CD74^+^ macrophages, we developed a novel delivery system by encapsulating Lut into ROS‐responsive PLGA NPs and functionalizing the surface of PLGA NPs with a MIF‐mimetic peptide sequence (MIF^79‐86^) which specifically binds to the extracellular domain of CD74, thereby enhancing the recognition and uptake of the NPs by CD74^+^ macrophages (**Figure** **6**A,B). The design strategy integrated multiple considerations for optimal therapeutic efficacy. First, the MIF‐mimetic peptide modification was implemented to replace the native MIF‐CD74 interaction, thereby facilitating specific recognition and enhanced uptake of nanocarriers by CD74^+^ macrophages. Second, MIF^79‐86^ competitively binds to CD74 and blocks the MIF signaling pathway in CD74^+^ macrophages. Third, the incorporation of ROS‐responsive diselenide bonds into the PLGA carrier structure recognizes the elevated ROS levels in inflammatory macrophages within the OA microenvironment.

The successful synthesis of the MDSPL delivery system was confirmed through comprehensive nano‐characterization. Transmission electron microscopy (TEM) and Dynamic light scattering (DLS) analysis showed that the hydrodynamic diameter of MDSPL increased from 141.3 ± 4.5 to 152.1 ± 2.9 nm upon functionalization with MIF^79‐86^ (Figure 6C,D). To evaluate the ROS‐responsive Lut release characteristics, we conducted comprehensive in vitro release analyses under various conditions. The release profile of Lut from MDSPL was assessed under physiological conditions and increasing concentrations of hydrogen peroxide (0, 0.01, 0.1, and 1 mm) to simulate varying levels of oxidative stress. Under physiological conditions (pH 7.4), the system demonstrated minimal Lut leakage, indicating excellent stability during circulation. In contrast, exposure to elevated hydrogen peroxide concentrations triggered accelerated Lut release in a concentration‐dependent manner, accompanied by material cracking and an increase in particle size. Furthermore, the release efficiency of Lut was found to be influenced by the temporal accumulation of ROS, leading to an expansion in the material volume of the MDSPL NPs. This volume expansion facilitated a faster release of Lut under fixed hydrogen peroxide concentrations (Figure 6E–H). This ROS‐dependent release behavior was attributed to the oxidative cleavage of diselenide bonds, resulting in controlled carrier degradation and Lut release. To assess the stability of MDSPL, the NPs were incubated in serum‐containing media at 37 °C, and their size was monitored over time. MDSPL MaDSPL maintained stability in size distribution for up to 7 days, indicating its excellent colloidal stability under physiological conditions (Figure 6I). UV–vis spectrophotometry analysis identified Lut's characteristic absorption maximum at 351 nm, which was used as the basis for subsequent quantitative analyses of Lut loading and release kinetics (Figure 6J). The Lut loading and encapsulation efficiency in DSPL were optimized at a ratio of 20:1 by adjusting the weight ratio of DS‐PLGA to Lut (Figure 6K).

### 2.6 MDSPL NPs Exhibit Enhanced Cellular Uptake and Exert Anti‐Inflammatory Effects in CD74^+^ Macrophages

**Figure 7:**
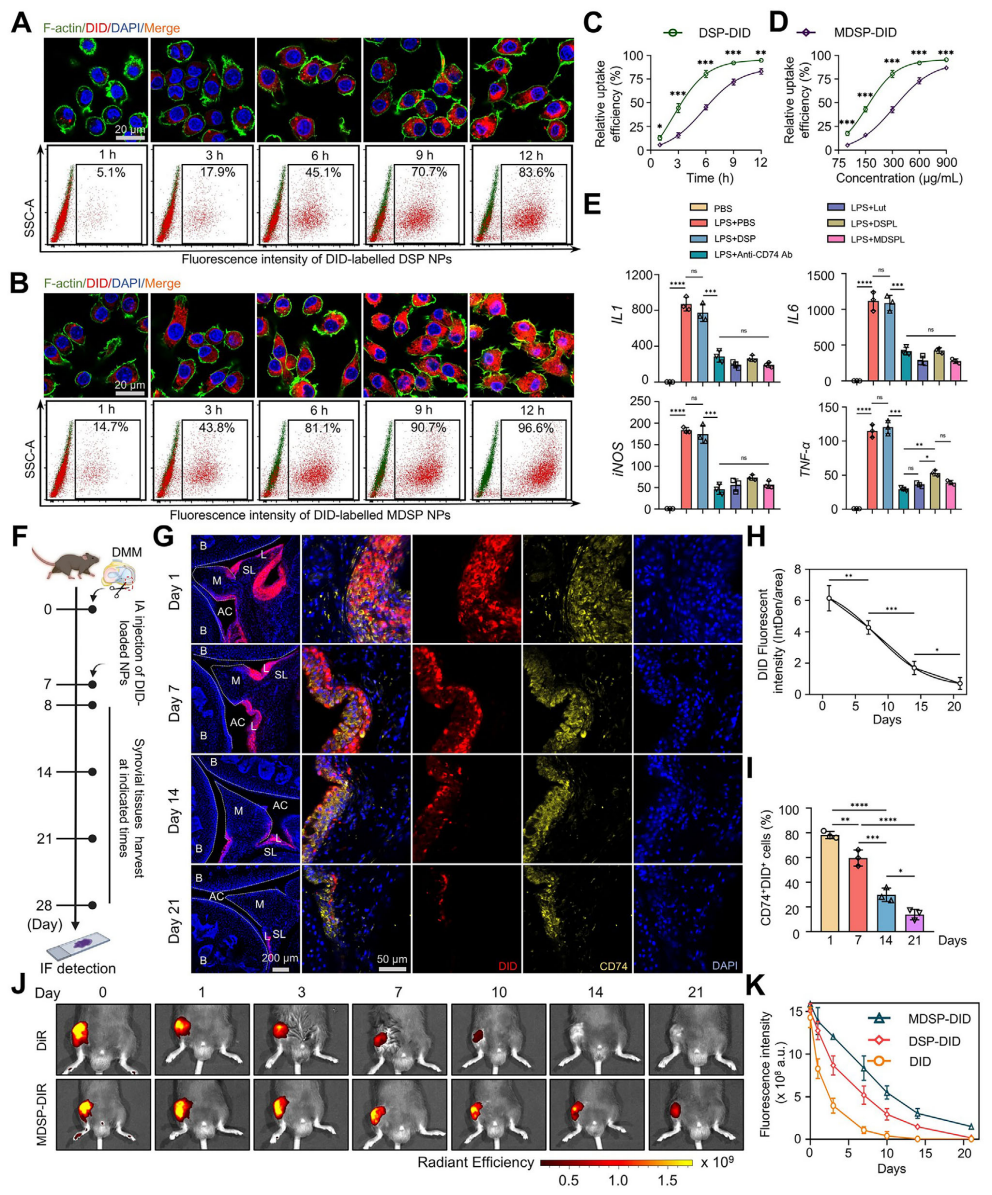
MDSPL NPs exhibit enhanced cellular uptake and exert anti‐inflammatory effects in CD74+ macrophages. A,B) Time‐dependent cellular uptake of DSP‐DiD and MDSP‐DiD NPs assessed by CLSM (upper panel) and FACS (lower panel). CD74+ macrophages were simultaneously treated with DSP‐DiD and MDSP‐DiD NPs for 1, 3, 6, 9, and 12 h, while the concentration of DID is approximately equal to 30 ug mL^−1^. Scale bar: 20 µm. C,D) The uptake efficiency of the DSP‐DiD and MDSP‐DiD NPs by CD74+ macrophages at specific time points was analyzed by the nonlinear fitting curve (*n* = 3 independent biological replicates at each pointed time). E) The mRNA expression levels of *IL1*, *IL6*, *iNOS*, and *TNF‐α* in RAW 264.7 macrophages treated by Lut and different formulations of NPs with or without LPS stimulation (*n* = 3 independent biological replicates per group). F) Schematic diagram of in vivo uptake evaluation of NPs by synovial tissues in mice subjected to DMM surgery. G,H) Fluorescence detection in joints of mice at different time points after injection of fluorescence system and metabolic assessment of nano‐delivery systems (B: bone; M: meniscus; AC: articular cavity; L: lining layer; SL: sublining layer) (*n* = 3 independent biological replicates per group). I) Quantitative statistics analysing the percent of CD74^+^DID^+^ macrophages in synovial tissues of mouse at different times (*n* = 3 independent biological replicates per group). J) Fluorescence localization detection in joints of mice at different time points after IA injection of DiR‐loaded NPs. K) Quantitative analysis of fluorescence intensity at different time points after intra‐articular injection of DiR‐labeled NPs (*n* = 3 independent biological replicates per group). The data are presented as mean ± SD. P values were calculated by (C‐D) two‐way ANOVA and (E‐F and J‐K) one‐way ANOVA. *P < 0.05, **P < 0.01, ***P < 0.001, ****P < 0.0001.

Importantly, Lut‐loaded formulations at concentrations up to 40 µg mL^−1^ exhibited no cytotoxicity in macrophages (Figure S8A, Supporting Information). To rigorously assess the targeting specificity and therapeutic efficacy of the MDSPL system toward CD74⁺ macrophages, flow cytometry was first employed to isolate the CD74⁺ subpopulation from LPS‐activated RAW 264.7 cells (Figure S8B, Supporting Information). Cellular uptake studies using the fluorescent probe DiD (incorporated into NPs as MDSP–1,1′‐dioctadecyl‐3,3,3′,3′‐tetramethylindodicarbocyanine, 4‐chlorobenzenesulfonate salt (DiD) to indirectly trace Lut‐containing formulations) demonstrated markedly enhanced internalization of peptide‐modified NPs compared with unmodified NPs, as shown by confocal laser scanning microscopy (CLSM) across time points (1–12 h, total material concentration: 600 µg mL^−1^, corresponding to 30 µg mL^−1^ of Lut) and multiple concentrations (75–900 µg mL^−1^, total material concentration). Flow cytometric quantification confirmed that MDSP–DiD uptake increased in both a time‐ and dose‐dependent manner (**Figure** **7**A–D; Figure S8C,D, Supporting Information), highlighting the contribution of MIF^79–86^/CD74 recognition to the targeting specificity. Building on this, functional assays demonstrated that Lut‐loaded NPs significantly downregulated *iNOS*, *TNF‐α*, *IL1*, and *IL6* expression in LPS‐stimulated macrophages, thereby validating their in vitro anti‐inflammatory efficacy (Figure 7E; Figure S8E, Supporting Information). In vivo, synovial tissue analysis following intra‐articular injection of DiD‐labeled NPs into OA mouse joints revealed that MDSP–DiD displayed robust localization within the synovium, with retention lasting up to 21 days (Figure 7F–H). Immunostaining further confirmed strong co‐localization of MDSP–DiD with CD74⁺ macrophages (Figure 7I), underscoring the specificity of the MIF‐mimetic peptide modification. To evaluate long‐term biodistribution, 1,1′‐dioctadecyl‐3,3,3′,3′‐tetramethylindocarbocyanine perchlorate (DiR)‐labeled MDSPL formulations were injected intra‐articularly into OA mouse knees. Compared to unmodified NPs, MDSP–DiR exhibited significantly prolonged intra‐articular retention, with detectable signals persisting for up to 21 days, whereas unmodified NPs were nearly cleared by day 10 (Figure 7J–K; Figure S9A,C, Supporting Information). Ex vivo fluorescence imaging of major organs at day 1 post‐injection demonstrated predominant liver accumulation, consistent with reticuloendothelial clearance pathways (Figure S9B,D, Supporting Information). Collectively, these findings establish that MDSPL not only ensures biocompatibility but also achieves superior synovial retention, controlled drug release, and enhanced targeting of CD74⁺ macrophages within inflamed joints, thereby positioning it as a promising nanomedicine platform for long‐acting, site‐specific therapy in osteoarthritis.

### 2.7 MDSPL NPs Alleviate OA‐Associated Pain and Cartilage Damage In Vivo

**Figure 8:**
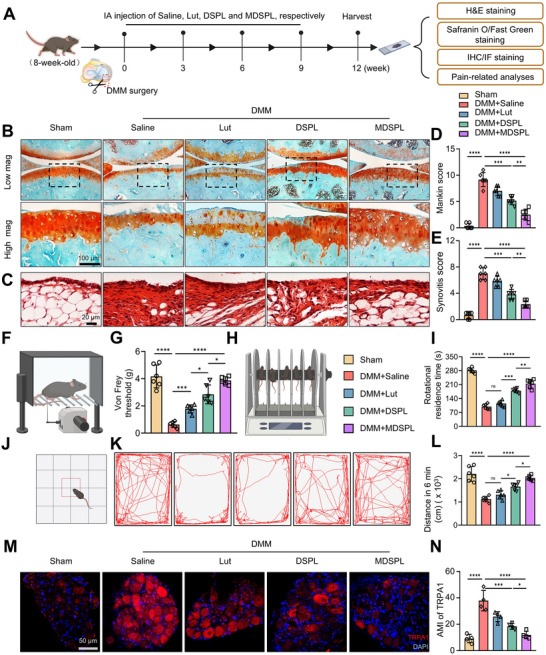
MDSPL NPs alleviate OA‐associated pain and cartilage damage in vivo. A) Schematic diagram illustrating the establishment of the OA mice model and the therapeutic evaluation of indicated treatments. B,C) Representative Safranin O/Fast green (Scale bar: 100 µm) and HE staining images (Scale bar: 20 µm) of knee joints at 12 weeks after DMM surgery. D,E) Quantitative assessment of OA severity with Mankin scores and synovial scores (*n* = 6 independent biological replicates per group). F–I) Comprehensive behavioral analyses including mechanical allodynia assessment via von Frey testing (F‐G) and motor function evaluation through rotarod performance (H‐I) prior to tissue harvest (*n* = 6 independent biological replicates per group). J–L) Open field test analysis of mice movement including a detailed visualization of the exploratory behavior and cumulative distance traveled by the mice over time (*n* = 6 independent biological replicates per group). M,N) Representative fluorescence images and AMI quantification of TRPA1 in DRG tissues of mice from different groups at 12 weeks after DMM modeling (*n* = 4 independent biological replicates per group). The data are presented as mean ± SD. P values were calculated by (D‐E, I, Land N) one‐way ANOVA. *P < 0.05, **P < 0.01, ***P < 0.001, ****P < 0.0001.

**Figure 9:**
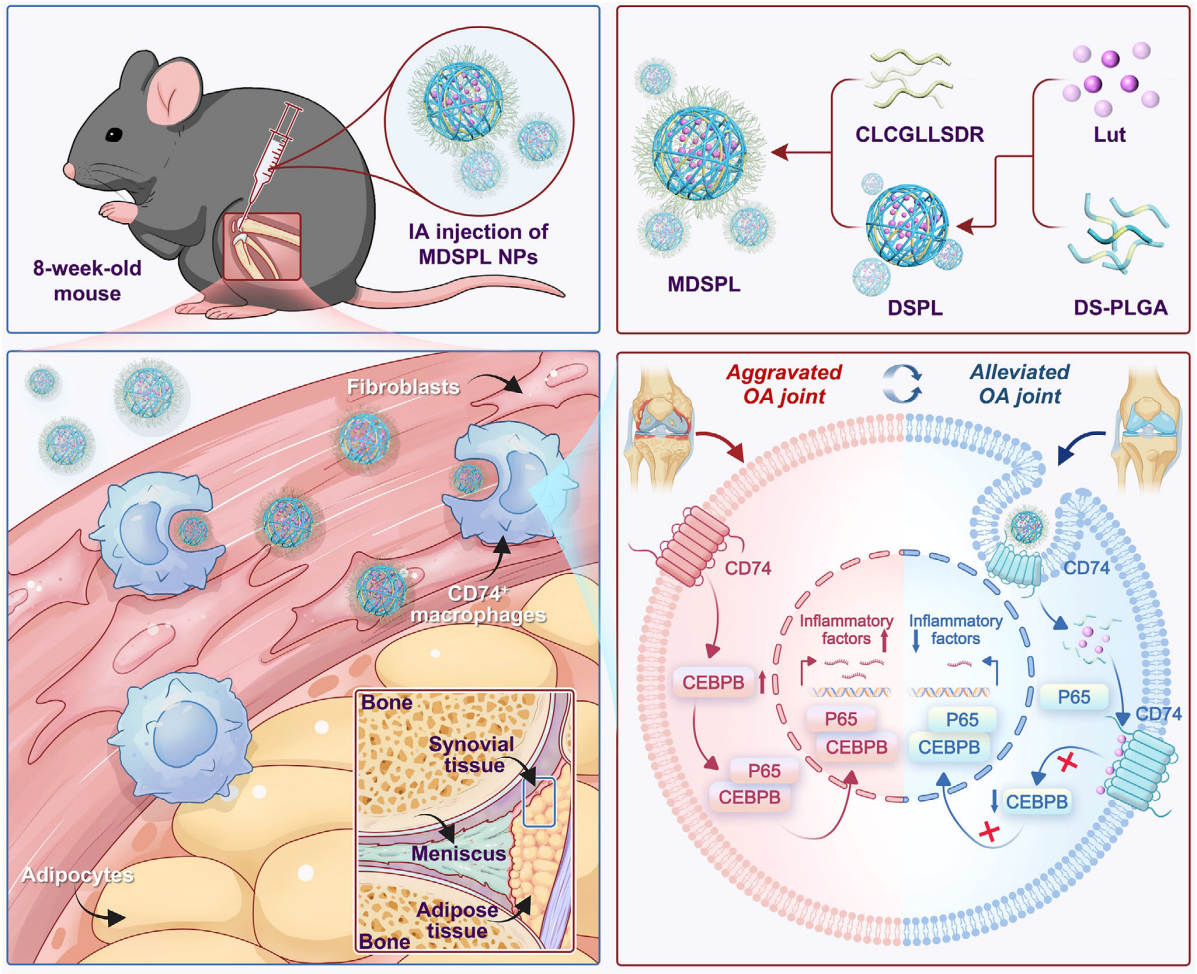
Schematic illustration of MDSPL NPs for targeted delivery of Lut to CD74^+^ macrophages in OA treatment. i) The DS‐PLGA NPs were engineered to encapsulate Lut and surface‐modified with MIF‐mimetic peptide sequences to target CD74**^+^** macrophages. ii) MDSPL was administered via IA injection into the OA joint of mouse induced by DMM surgery. iii) The MIF‐mimetic peptides on MDSPL surface specifically recognize and bind to CD74 receptors highly expressed on CD74**+** macrophages, facilitating efficient cellular internalization through CD74‐mediated endocytosis. ix) Following cellular internalization, the elevated ROS levels in inflammatory macrophages trigger the cleavage of diselenide bonds, leading to Lut release. The released Lut binds to the intracellular domain of CD74, subsequently downregulating CD74 expression. This downregulation reduces CEBPB levels and disrupts its interaction with P65, thereby inhibiting P65 nuclear translocation and NF‐κB pathway activation. The suppression of this inflammatory cascade ultimately attenuates macrophage‐mediated inflammation and ameliorates OA progression.

The therapeutic efficacy of MDSPL was evaluated in a DMM‐induced OA murine model, with dosing intervals informed by our biodistribution findings showing that peptide‐modified NPs localized predominantly in synovial tissues and persisted for up to 21 days post‐injection (Figure 7F–K). Accordingly, intra‐articular administration was performed every 3 weeks over a 12‐week treatment course, ensuring both alignment with the pharmacokinetic profile of the NPs and the successful establishment of OA pathology (**Figure** **8**A). At 12 weeks post‐surgery, saline‐treated mice exhibited severe cartilage destruction characterized by erosion, loss of superficial and intermediate layers, hypertrophic chondrocytes within the calcified zone, and significantly elevated Mankin and synovitis scores. In contrast, free Lut or DSPL treatment partially preserved tibial cartilage architecture, though surface fibrillation, clefts, and reduced safranin O staining were evident, accompanied by chondrocyte hypertrophy. Strikingly, MDSPL treatment resulted in nearly intact articular cartilage with only minimal fibrillation and mild loss of matrix staining, which was reflected in significantly lower Mankin and synovitis scores relative to saline‐treated groups (Figure 8B–E). Functionally, MDSPL also alleviated OA‐associated pain and improved motor performance, as evidenced by reduced mechanical allodynia in the von Frey assay, enhanced endurance on the rotarod test, and greater locomotor activity in the open field test, where total distance traveled was significantly increased (Figure 8F–L). At the mechanistic level, MDSPL treatment reduced transient receptor potential ankyrin 1 (TRPA1) expression in dorsal root ganglion neurons, consistent with its analgesic effects (Figure 8M,N).^[^ ^26^ ^]^ Immunofluorescence and flow cytometry analyses further revealed that both DSPL and MDSPL attenuated synovial macrophage inflammation by downregulating MMP13, iNOS and IL6 expressions, with MDSPL producing more pronounced effects (Figure S10A–C,G–I, Supporting Information). Moreover, MDSPL markedly suppressed the expression of CD74, CEBPB, and P65 in synovial macrophages, indicating inhibition of the CD74/CEBPB/NF‐κB axis, again with stronger effects than unmodified NPs (Figure S10D–F,J–L, Supporting Information). Histological analyses of vital organs (heart, liver, spleen, lung, and kidney) confirmed the absence of systemic toxicity across all treatment groups (Figure S11, Supporting Information). Importantly, when treatment was delayed until four weeks post‐DMM surgery to mimic clinical presentation (Figure S12A, Supporting Information), MDSPL retained partial efficacy but exhibited attenuated cartilage‐protective and anti‐inflammatory effects compared with immediate intervention (Figure S12B–E, Supporting Information), highlighting the importance of early therapeutic administration. Collectively, these findings demonstrate that MDSPL substantially mitigates OA progression by targeting CD74⁺ macrophages, disrupting the CEBPB–p65 complex formation and preventing p65 nuclear translocation, thereby suppressing synovial inflammation, preserving cartilage, and alleviating pain, with superior efficacy compared to free Lut or unmodified NPs. These results establish MDSPL as a promising nanomedicine strategy for OA treatment, particularly in the context of early intervention (**Figure** **9**).

## 3 Discussion

OA represents a complex chronic inflammatory disease.^[^ ^27^, ^28^ ^]^ Notably, synovial inflammation, predominantly driven by macrophages, which are major sources of inflammatory cytokines and chemokines, may precede cartilage degeneration in OA progression, highlighting its crucial role in disease development and potential therapeutic targeting.^[^ ^29^, ^30^ ^]^ By systematically integrating scRNA‐seq transcriptomics, our evaluation of clinical end‐stage OA patients' synovial specimens has led to the identification and validation of a distinct CD74^+^ macrophage subpopulation with pronounced inflammatory properties.

Building on our initial scRNA‐seq findings, we sought to further delineate the characteristics of the CD74⁺ macrophage subpopulation within the OA synovial microenvironment. Immunofluorescence analysis demonstrated the co‐localization of CD74 with F4/80, thereby confirming their macrophage identity. Importantly, these CD74⁺ macrophages exhibited enhanced co‐expression of pro‐inflammatory mediators, including MMP13 and iNOS, in OA synovial tissues compared with normal controls, providing strong evidence of their inflammatory phenotype. Notably, our analyses revealed that CD74⁺ macrophages cannot be fully classified within the framework of traditional M1/M2 polarization. While they share several pro‐inflammatory features with classically activated M1 macrophages, they also display unique transcriptional signatures and functional properties that distinguish them from conventional subsets. These observations suggest that CD74⁺ macrophages represent a distinct pathogenic subset within the synovium. More broadly, our findings support the concept that macrophage heterogeneity in OA exists along a dynamic spectrum of activation states, rather than conforming strictly to the binary M1/M2 paradigm. This redefinition of macrophage plasticity highlights the pathological relevance of the CD74⁺ subset and underscores its potential as a therapeutic target in OA. In addition, MIF is a well‐established ligand of CD74 and has been shown to play a critical role in OA pathogenesis. Elevated MIF levels have been consistently detected in both the serum and synovial fluid of OA patients, indicating its involvement in disease progression.^[^ ^31^ ^]^ In vivo studies further support this role: genetic deletion of MIF in mice conferred protection against age‐related OA, resulting in reduced cartilage degradation, attenuated synovial hyperplasia, and mitigated subchondral bone remodeling compared to controls.^[^ ^31^ ^]^ Our single‐cell transcriptomic analysis revealed a distinct compartmentalization of the MIF–CD74 signaling axis within the OA synovium. Macrophages constituted the predominant CD74‐expressing population, whereas fibroblasts—particularly platelet‐derived growth factor receptor alpha (PDGFRα⁺) subsets—served as the principal source of MIF. This cell‐type–specific paracrine interaction suggests that fibroblast‐derived MIF may activate CD74⁺ macrophages, thereby driving the initiation and amplification of synovial inflammation during osteoarthritis progression. These works highlight the pathogenic significance of the MIF–CD74 axis in OA and reinforce the rationale for targeting this signaling pathway as a potential therapeutic strategy to modulate macrophage‐driven synovial inflammation and joint degeneration.

In addition to identifying and characterizing a distinct CD74⁺ macrophage subpopulation with pronounced inflammatory properties in OA synovitis, we further demonstrated that Lut, a natural flavonoid compound, exerts a potent regulatory effect on this subset through reverse pharmacological screening. The rational selection of Pocket_1 for virtual screening, based on its superior druggable landscape, was critical to this discovery. While several top‐ranking compounds were identified, Lut emerged as the most promising candidate not merely due to its superior docking score, but more importantly, because its predicted binding mode involved a greater number of specific hydrogen‐bond interactions within the pocket. This structural insight suggests a more stable and energetically favorable binding mechanism compared to other hits. Consistent with these predictions, SPR confirmed Lut's strong binding to CD74, and it exhibited promising efficacy in subsequent assays. These results validate both the sensitivity and reliability of our docking methodology. While the inherent simplifications of molecular docking are acknowledged, the combination of in silico modeling and empirical verification effectively addressed these limitations. Our study identifies Lut as a validated lead compound targeting CD74.

The integrated transcriptomic analyses from Lut‐treated and CD74‐knockout macrophages, combining pharmacological intervention with genetic manipulation, revealed that CEBPB functions as a pivotal downstream effector of the Lut/CD74 axis and uncovered the involvement of the NF‐κB signaling pathway. CEBPB is a well‐established transcription factor implicated in diverse inflammatory diseases, including rheumatoid arthritis and inflammatory bowel disease, where it regulates key inflammatory mediators.^[^ ^32^, ^33^, ^34^ ^]^ However, its contribution to macrophage‐driven inflammation during OA progression has remained poorly defined. In this study, we demonstrate markedly elevated CEBPB expression in both human and murine OA synovial tissues, with pronounced co‐localization in CD74⁺ macrophages, thereby establishing a functional relationship between CD74 and CEBPB in orchestrating inflammatory responses. This spatial association, consistently observed across species, suggests that CEBPB activation represents a conserved mechanism underlying CD74‐dependent inflammation. Furthermore, while prior studies have alluded to potential interactions between CEBPB and the NF‐κB pathway component p65, the upstream regulatory context has remained elusive. Our findings significantly advance this understanding by showing that CD74 activation under inflammatory conditions promotes the interaction of CEBPB with p65, facilitating p65 nuclear translocation and thereby amplifying NF‐κB signaling. Collectively, these results delineate a mechanistic signaling axis in which CD74 overexpression drives synovial inflammation through CEBPB‐mediated activation of the NF‐κB pathway. By linking CD74, CEBPB, and NF‐κB into a coherent regulatory cascade, this study provides novel insights into the molecular basis of CD74^+^ macrophage‐mediated inflammation in OA and identifies potential therapeutic targets for modulating synovial pathology.

To achieve targeted delivery of Lut to CD74^+^ macrophages, we adopted a distinct strategy by harnessing the intrinsic affinity of MIF for CD74 to achieve targeted drug delivery. Specifically, to address the limitations of small‐molecule compounds such as Lut—including rapid clearance, poor stability, and restricted bioavailability—we engineered a novel nanoparticle platform by functionalizing DS‐PLGA nanoparticles with MIF‐mimetic peptide sequences. This modification enabled selective recognition and uptake of Lut‐loaded MDSPL nanoparticles by CD74⁺ macrophages within the synovial microenvironment.

Comparative analyses demonstrated that MDSPL exhibited superior anti‐inflammatory efficacy relative to free Lut, both in vitro and in vivo. The advantages of this system were evident in several aspects: first, enhanced intra‐articular retention prolonged therapeutic activity; second, targeted delivery to CD74⁺ macrophages minimized off‐target effects while maximizing local efficacy; and third, the ROS‐responsive controlled release profile of DS‐PLGA ensured sustained drug availability, thereby reducing dosing frequency. These features translated into markedly improved therapeutic outcomes in experimental OA models, characterized by reduced cartilage degradation, attenuated synovial inflammation, and alleviated pain. These findings underscore the potential of exploiting MIF–CD74 interactions not only as a therapeutic target but also as a versatile platform for precision nanomedicine in osteoarthritis.

The therapeutic promise of MDSPL is reinforced by its excellent safety profile. Comprehensive biocompatibility evaluations demonstrated > 95% macrophage viability at therapeutic concentrations, indicating minimal cytotoxicity and preserving the functional integrity of resident immune cells. In vivo assessments further confirmed systemic safety, as no histopathological abnormalities were observed in major organs after a 12‐week intra‐articular treatment regimen. These results highlight the intrinsic biocompatibility of the PLGA‐based delivery system and validate both local and systemic tolerability of MDSPL. Given the absence of approved anti‐inflammatory drugs specifically for OA, an anti‐CD74 antibody was employed as a positive control. The comparable anti‐inflammatory efficacy observed between the antibody and both free Lut and its nano‐formulations provides strong evidence that the therapeutic effects are CD74‐dependent. The convergence of cytokine suppression profiles from two mechanistically distinct agents—a small molecule and a monoclonal antibody—confirms CD74 as the principal target of Lut. Moreover, the inactivity of blank DSP nanoparticles verifies that the observed effects are drug‐specific rather than carrier‐induced. These findings establish CD74 as a pivotal regulator in macrophage‐mediated inflammation.

Despite the substantial advances achieved in this study, several limitations merit consideration when interpreting the findings and planning future research. First, the precise epigenetic mechanisms through which CD74 regulates CEBPB expression remain incompletely defined and require further elucidation to fully understand the upstream control of this axis. Second, although our data establish synovial tissue and macrophages as principal sources of inflammatory mediators in OA, the dynamic and reciprocal interactions between these cellular populations in driving disease progression remain insufficiently characterized. Third, while the therapeutic efficacy of MDSPL nanoparticles was validated in murine models, comprehensive long‐term safety evaluations, including biodistribution and toxicity studies in larger animals, are essential to assess systemic effects and ensure translational feasibility. In addition, OA is a degenerative disease, the aging process itself profoundly shapes macrophage phenotypes and inflammatory responses, future work would focus on the effect of aging on macrophage CD74 expression within the synovium using scRNA‐seq. These limitations notwithstanding, our study provides a solid foundation for future investigations. The identification of the CD74–CEBPB–P65 axis as a critical mediator of macrophage‐driven inflammation, together with the development of a CD74‐targeted nanoparticle delivery system, represents an important step forward in advancing OA therapeutics. Addressing these outstanding questions through mechanistic and translational studies will not only strengthen the biological understanding of OA pathogenesis but also accelerate the clinical development of precision nanomedicine strategies for OA and potentially other inflammatory joint diseases.

## 4 Experimental Section

### Experimental Design

This study was structured to systematically investigate the role of CD74 in OA‐associated synovial inflammation and to develop a targeted therapeutic strategy. The experimental framework comprised four integrated phases: i) A synovial single‐cell atlas was compiled using scRNA‐seq of mouse synovial tissue. Specific macrophage subpopulations were identified, revealing a CD74^+^ macrophage subset with pro‐inflammatory characteristics that may contribute to OA progression. ii) Network pharmacology was employed to identify small molecules regulating CD74 expression, with Lut emerging as a candidate. The regulatory effect of Lut on CD74 expression and its therapeutic potential for OA were validated through in vitro and in vivo experiments. iii) By combining pharmacological inhibition of CD74 with CD74 genetic knockout models, RNA‐seq analysis revealed that Lut modulates CEBPB expression, suppresses P65 nuclear translocation, and inhibits NF‐κB pathway activation, thereby mitigating macrophage‐driven inflammation. iv) A PLGA‐based nanoparticle drug delivery system was developed, incorporating Lut and a surface‐modified peptide mimicking MIF protein. This peptide specifically binds the extracellular domain of CD74, enabling targeted delivery and exogenous modulation of CD74 expression in synovial tissue. All animal cohorts (minimum *n* = 3 per group) and in vitro studies (≥ 3 biological replicates) followed randomized allocation. No data points were excluded. Statistical analyses were blinded to group identity. Sample sizes for individual experiments were specified in the corresponding figures.

### Acquisition and Processing of scRNA Data

Publicly available synovial scRNA‐seq datasets were retrieved from the Gene Expression Omnibus (GEO) database. Human OA synovial data (GSE216651) were derived from a study by Su'an Tang et al.,^[^ ^3^ ^]^ in which synovial tissues were obtained from end‐stage knee OA patients (Kellgren‐Lawrence grade ≥3) undergoing total knee replacement surgery at Zhujiang Hospital of Southern Medical University, China. Mouse OA synovial data (GSE231755) originated from the previous work,^[^ ^35^ ^]^ where synovial tissues were harvested from *WT* C57BL/6J mice subjected to DMM surgery. Mice were stratified into three groups: control (2‐month‐old, *n* = 20), 1‐week post‐DMM (*n* = 8), and 2‐month post‐DMM (*n* = 12). These datasets comprised 24354 cells from synovial tissues of OA mice, as well as 10605 cells from synovial tissues of 3 OA patients. Raw count expression matrices were processed using the Seurat R package. The Seurat R package (version 5.0.3) was used to import and filter the single‐cell data. Stringent quality control criteria were applied, including thresholds based on mitochondrial gene percentage and gene detection depth. A normalized expression matrix containing 3000 highly variable genes was generated. Dimensionality reduction was performed using the first 30 principal components (PCA) in combination with the Harmony package (version 1.2.0) to mitigate batch effects. Marker genes were identified using the FindAllMarkers function and subsequently used for clustering and visualization. 2D clustering analysis was performed using Uniform Manifold Approximation and Projection (UMAP) to delineate and characterize high‐resolution cell clusters. Normalization and scaling were conducted using the SCTransform function to reduce the influence of cell cycle effects. To integrate datasets and remove batch effects, the “SCT” normalization method was applied, with the dimensionality reduction parameter set to 20. Clustering and dimensionality reduction were performed using the KNN‐based algorithm. These preprocessing steps ensured high‐quality data for downstream analysis. Trajectory analysis of CD74^+^ macrophages was conducted using Monocle 2 to reconstruct cellular state transitions. The reversed graph embedding algorithm was applied to infer single‐cell trajectories.

### Human Synovial Tissue Isolation and Cohort Selection Criteria

Ethical approval for this study was obtained from the Institutional Review Board of the First Affiliated Hospital of Jinan University (Approval No. MR‐44‐23‐050188), and all participants provided written informed consent. Synovial tissues were prospectively collected from two cohorts: 1) OA cohort: Patients with end‐stage knee osteoarthritis (Kellgren‐Lawrence grade ≥3) undergoing primary total knee replacement surgery (*n* = 8; median age: 71.2 years; 62.5% female); 2) Control cohort: Individuals with arthroscopic procedures for traumatic meniscal tears or anterior cruciate ligament reconstruction (*n* = 5; median age: 27.1 years; 60% male). Exclusion criteria included: i) autoimmune diseases (e.g., rheumatoid arthritis, psoriasis); ii) infectious arthritis; iii) prior intra‐articular steroid injection within 6 months; iv) malignancy; or v) systemic inflammatory conditions.

Synovial specimens were harvested within 30 min of surgical excision, divided into aliquots for histopathology (fixed in 4% paraformaldehyde) or snap‐frozen in liquid nitrogen (protein extraction). Histopathological validation included H&E staining and immunohistochemistry.

### Synovial Explants Isolation and Culture

The synovial tissues were cut into standardized explant dimensions of ≈5 mm (length) × 5 mm (width) × 3 mm (depth). Three independent tissue explants were derived from each donor, and experiments were repeated three times (biological replicates = 3 donors). The tissue explants were then placed in a culture medium composed of DMEM supplemented with 1% Insulin‐Transferrin‐Selenium (ITS, MCE, HY‐150287, USA), 50 µg mL^−1^ L‐proline (MCE, HY‐Y0252, USA), 0.1 µm dexamethasone (MCE, HY‐14648, USA), 0.9 mm sodium pyruvate (Gibco, 11360‐070, USA), and 50 µg mL^−1^ ascorbic acid 2‐phosphate (MCE, HY‐103701, USA). The explants were allowed to acclimatize in this medium for 2 days at 37 °C in a humidified atmosphere of 5% CO_2_. After the initial acclimatization period, the synovial explants were randomly treated with PBS, IL‐1β recombinant protein (10 ng mL^−1^, PeproTech, 200‐01B‐1MG, USA) and IL‐1β recombinant protein combined with Lut. The treatment medium was refreshed every 2 days to maintain consistent concentrations of IL‐1β and Lut. After 8 days of treatment, the synovial explants were fixed in 4% paraformaldehyde, embedded in paraffin, and sectioned for subsequent histological and immunohistochemical analyses. IF‐stained sections were imaged using a confocal microscope (Zeiss LSM 880).

### Cell Culture and Primary Cell Isolation

RAW 264.7 cells (SCSP‐5036, CHINA) were obtained from the Cell Bank of the Chinese Academy of Sciences and maintained in Dulbecco's modified Eagle's medium (DMEM, Gibco, C12430500BT, USA) supplemented with 10% heat‐inactivated fetal bovine serum (FBS, Gibco, 10 091 148, USA) and 1% penicillin‐streptomycin (PS, Gibco, 15 140 148, USA) solution at 37 °C in a humidified atmosphere containing 5% CO_2_. Bone marrow cells were isolated from the femurs of 8‐week‐old wild‐type (*WT*) mice. Following red blood cell lysis using ACK lysis buffer (Beyotime, C3702, CHINA), bone marrow cells were collected and centrifuged at 300 × g for 5 min. The cell pellet was resuspended in α‐Minimum Essential Medium (α‐MEM, Gibco, C12571500BT, USA) supplemented with 10% heat‐inactivated FBS, 1% PS solution, and 30 ng mL^−1^ mouse macrophage colony‐stimulating factor (M‐CSF, MCE, HYP70553, USA) to generate mature BMDMs. For inflammatory stimulation, macrophages were treated with LPS (Beyotime, S1732, CHINA) at a final concentration of 50 ng mL^−1^ for 24 h.

Primary chondrocytes were isolated from 1‐week‐old *WT* mice. Briefly, ≈0.5 cm of translucent cartilage tissue between the tibial and femoral growth plates was harvested from the knee joints. The tissue was minced and digested with 0.25% trypsin (Thermo, 25 200 072, USA) for 30 min, followed by digestion with type I collagenase (2 mg mL^−1^, Thermo, 17 100 017, USA) for 3 h at 37 °C with gentle agitation. The digestion was terminated by adding complete culture medium. After filtration, the cell suspension was centrifuged, and the resulting cell pellet was resuspended in DMEM supplemented with 10% heat‐inactivated FBS and 1% PS solution.

### Isolation and Characterization of Synovial Macrophages

DMM surgery was performed on 8‐week‐old male mice, and synovial tissues were harvested at 7 days post‐surgery. The synovial tissues were collected into 15 mL tubes and digested sequentially with 0.25% trypsin‐EDTA (Gibco, 25 200 056, USA) solution and 300 U mL^−1^ collagenase type I (MCE, HY‐E70005A, USA) to obtain a single‐cell suspension. The cell suspension was stained with a specific antibody to distinguish CD74 positive (CD74⁺) and CD74 negative (CD74^−^) macrophages. Flow cytometry staining was performed using antibodies against CD45 (Biolegend, 147 719, USA), CD11b (Biolegend, 101 206, USA), CD74 (Biolegend, 151 004, USA) and F4/80 (Biolegend, 111 604, USA) following the manufacturer's protocol. Cell sorting was conducted using a fluorescence‐activated cell sorting (FACS) Aria flow cytometer (BD Biosciences) to isolate F4/80^+^CD74⁺ and F4/80^+^CD74^−^ macrophages. Subsequently, RNA was extracted from the sorted cells, and qPCR was performed to assess the expression levels of key molecular markers associated with F4/80^+^CD74⁺ and F4/80^+^CD74^−^ macrophages.

### Identification and Virtual Screening of CD74‐Binding Compounds

A comprehensive in silico screening approach was implemented to identify potential CD74‐targeting compounds. Given the absence of crystallographic data for CD74, AlphaFold2 (v2.0) was first employed for protein structure prediction. The predicted model underwent energy minimization using the AMBER force field, followed by binding pocket analysis using DoGSite3 (v3.0.1) to identify potential ligand‐binding sites. Candidate compounds were initially identified through the Coremine Medical database based on reported CD74 interactions. Chemical structures and properties were retrieved from TCMSP and PubChem databases. The collected compounds were filtered based on pharmacokinetic properties, including absorption, distribution, metabolism, and excretion (ADME) characteristics. The filtered compounds underwent structural optimization using Open Babel (v3.1.1) for energy minimization and protonation state assignment at physiological pH. Molecular docking was performed using AutoDock Vina (v1.2.0). Pymol was utilized to evaluate and visualize the binding interactions between CD74 and the top‐ranked compound.

### CD74 Knockdown and CEBPB Overexpression

The siRNAs targeting CD74 were synthesized by GenePharma (Shanghai, China). For transient knockdown experiments, cells were seeded in 6‐well plates (Corning Costar, 3516, USA) and cultured for 24 h to reach 60–70% confluence. Transfections were performed using Lipofectamine 3000 (Thermo, 11 668 019, USA) according to the manufacturer's protocol. Specifically, cells were transfected with either control siRNA (si‐NC) or CD74‐specific siRNA (si‐CD74) in 1 mL of serum‐free medium for 4 h. After transfection, the medium was replaced with a complete medium containing 10% FBS, and cells were further cultured for 72 h to ensure effective knockdown. The sequences of siRNAs used in this study, including si‐CD74 and si‐NC, are detailed in Table S3 (Supporting Information). The efficiency of CD74 knockdown was confirmed by qPCR and IB analysis.

To overexpress CEBPB, its CDS was amplified with designed primers and cloned it into the pKD‐EF1a‐HA vector via AscI and EcoRI sites (Figure S8 and Table S4, Supporting Information). The plasmid was then synthesized by PCR and ligation with recombinant enzymes and validated by sequencing. For transfection, 1 × 10⁶ macrophages were seeded in 15 cm dishes until 30–40% confluence. A mixture of 10 µg plasmid, 30 µL Lipofectamine 2000, and 1 mL opti‐DMEM was incubated for 15 min before addition. The medium was changed 8 h post‐transfection and was replaced with a complete medium containing 10% FBS for a further culture period of 48 h.

### Bulk RNA Sequencing

Total RNA was extracted from the DMSO‐ or Lut‐treated RAW 264.7 cells and *CD74^+/+^*/*CD74^−/−^* primary macrophages pre‐activated by LPS. RNA integrity was assessed using an Agilent 2100 Bioanalyzer (Agilent Technologies, Santa Clara, CA, USA). RNA sequencing libraries were constructed and sequenced on the Illumina NovaSeq 6000 platform (Illumina, San Diego, CA, USA) to generate high‐quality transcriptomic data. DEGs between different groups were identified using the DESeq2 R package. Genes with a false discovery rate (FDR) < 0.05 and |log2 fold change| ≥ 1 were considered statistically significant. Hierarchical clustering of DEGs was visualized as heatmaps using the Pheatmap package, while volcano plots and KEGG peak maps were generated using the ggplot2 package.

### Quantitative Real‐Time PCR (qPCR)

Total RNA was extracted from samples using TRIzol Plus reagent (Takara, 9109, JAPAN). Subsequently, 1 µg of purified RNA was reverse‐transcribed into complementary DNA (cDNA) using the High‐Capacity cDNA Reverse Transcription Kit (Trans, AT311‐03, CHINA) with slight modifications to optimize the reaction conditions. The expression levels of target genes, including *CD74*, *IL1*, *IL6*, *iNOS*, *TNF*‐α, *ATF3*, *PLK2*, *COL2A1*, *MMP13*, *CEBPB*, and *GAPDH*, were analyzed by qPCR. Primer sequences for the specific genes are listed in Table S5 (Supporting Information). Reactions were performed using a One Step Real‐Time PCR system (Applied Biosystems) and Fast SYBR GREEN Master Mix (Trans, AQ601‐02, CHINA). qPCR reactions were conducted in triplicate for each sample, and all experiments were repeated at least three times independently. The relative expression levels of target genes were normalized to the housekeeping gene GAPDH as an internal control. Gene expression differences were calculated using the comparative 2^−ΔΔCT^ method.

### Histology Analysis

Formalin‐fixed paraffin‐embedded tissue sections were deparaffinized in xylene and rehydrated through a graded ethanol series. Antigen retrieval was performed using Tris‐EDTA buffer (pH 9.0, Servicebio, G1206, CHINA) at 95 °C, followed by permeabilization with 0.5% Triton X‐100 for 15 min. Non‐specific binding was blocked by incubating the sections with 3% bovine serum albumin (BSA, Beyotime, ST023) for 1 h at room temperature. After blocking, the sections were incubated overnight at 4 °C with primary antibodies, including anti‐CD74 (1:200, Servicebio, GB115427, China), anti‐F4/80 (1:200, cell signaling technology (CST), 30 325, USA), anti‐iNOS (1:200, Servicebio, GB11119, China), anti‐MMP13 (1:200, Servicebio, GB11247, China), anti‐CD68 (1:200, CST, 97 778, USA), anti‐IL6 (1:200, Novus, NB600‐1131, USA), anti‐CEBPB (1:200, Proteintech, 23431‐1‐AP, China), anti‐TRPA1 (1:200, Abcam, ab2721, USA), anti‐P65 (1:200, CST, 8242, USA). Sections were then incubated with fluorescent dye‐conjugated anti‐rabbit/mouse IgG (H+L), F(ab')2 Fragment from CST (1:200) for 1 h at room temperature in the dark. Slides were mounted using 4′,6‐diamidino‐2‐phenylindole (DAPI) Fluoromount‐G mounting medium (SouthernBiotech, 0100‐20, USA). Images were captured using a Zeiss LSM 880 confocal microscope and processed with Zen 2021 software (Carl Zeiss Microimaging LLC). Quantification of fluorescence intensity and co‐localization was performed using ImageJ software. For each experimental group, 3 biological replicates (synovial tissues from independent donors) and 3 technical replicates per biological replicate were analyzed. Background fluorescence was subtracted using adjacent non‐stained areas. Co‐localization of target protein signals was quantified using Pearson's correlation coefficient (PCC) and Manders’ overlap coefficient (MOC). Only signals with significant co‐localization (PCC > 0.6) were considered macrophage‐specific.

HE staining and Safranin O/Fast Green staining were performed as previously described.^[^ ^36^ ^]^ The severity of OA was evaluated using the Mankin scoring system. Synovitis severity was assessed based on the following morphological parameters:^[^ ^37^ ^]^ i) synovial lining layer hyperplasia/expansion, ii) degree of inflammatory cell infiltration, and iii) activation of stromal cells (fibroblasts, endothelial cells, histiocytes, macrophages, and multinucleated giant cells). Each parameter was scored from 0 (none) to 3 (severe). Investigators were blinded to treatment groups during ROI selection and analysis.

### Immunoblotting (IB) Analysis

Briefly, protein samples (20 µg per lane) were separated by 10% sodium dodecyl sulfate‐polyacrylamide gel electrophoresis (SDS‐PAGE) (Beyotime, P0012A, China) and transferred onto polyvinylidene difluoride (PVDF) membranes. The membranes were blocked with 5% non‐fat milk at room temperature for 1 h to prevent non‐specific binding. After blocking, the membranes were incubated overnight at 4 °C with primary antibodies targeting CD74 (1:1000, Servicebio, GB115427, China), MMP13 (1:1000, Servicebio, GB11247‐1, China), iNOS (1:1000, Servicebio, GB125703, China), CEBPB (1:1000, Proteintech, 23431‐1‐AP, China), P65 (1:1000, CST, 8242, USA), COL2A1 (1:1000, Abcam, ab34712, USA), and IL6 (1:000, CST, 12912, USA). GAPDH was used as a loading control. The membranes were then incubated with horseradish peroxidase (HRP)‐conjugated secondary antibodies (1:1000, CST, 7074, USA) at room temperature for 1 h. Protein bands were detected using an enhanced chemiluminescence (ECL, Biorad, 1 705 060, USA) kit and visualized using an imaging system.

### Co‐immunoprecipitation (Co‐IP) Assay

Co‐IP was performed to investigate the endogenous interaction among CEBPB, CD74, and P65 in mBMDMs following standard protocols. Briefly, cells with or without stimulation were harvested and lysed by sonication in Western and IP Lysis Buffer (Beyotime, P0013, China) supplemented with sodium fluoride (NaF), phenylmethylsulfonyl fluoride (PMSF), sodium orthovanadate (Na_3_VO_4_), and a protease inhibitor cocktail. The lysates were incubated on ice for 30 min to ensure complete cell lysis. Next, 40 µL of a 50% protein A agarose bead slurry (CST, 9863, USA) was added to the lysates to preclear nonspecific binding proteins by incubation for 30 min at 4 °C with gentle agitation. Precleared lysates were then incubated overnight at 4 °C with primary antibodies, including anti‐CEBPB (1:50, Proteintech, 23431‐1‐AP, China), anti‐CD74 (1:50, Abcam, ab289885, USA), anti‐P65 (1:50, CST, 8242, USA) or control IgG (1:50, CST, 2729, USA). The antibody‐protein complexes were captured by incubating with 40 µL of the 50% protein A agarose bead slurry for an additional 4 h at 4 °C on a rotating platform. Immunoprecipitates were then washed five times with ice‐cold lysis buffer to remove nonspecific proteins. The immunoprecipitated proteins were eluted from the beads and subjected to IB analysis. To prevent interference from the IgG heavy chain, HRP‐conjugated secondary antibodies specific for the light chain (HRP AffiniPure Mouse Anti‐Rabbit IgG Light Chain, Abbkine, A25022, China) were used for detection.

### Murine OA Modeling Establishment

All animal procedures were approved by the Experimental Animal Ethics Committee of Jinan University and conducted in accordance with institutional guidelines (Approval Number: IACUC‐20241205‐04). Male *C57BL/6J* mice (8‐week‐old) were used to establish OA models via DMM surgery on the right knee. Briefly, the mice were anesthetized with isoflurane, and meloxicam was administered subcutaneously as an analgesic. The right knee joint area was shaved, disinfected, and prepared for surgery. A small longitudinal incision was made above the patellar ligament under sterile conditions, followed by careful dissection to expose the joint cavity. In the experimental group, the transverse ligament of the medial meniscus was completely transected using sterile microsurgical scissors, ensuring proper destabilization of the joint. For the sham surgery group, the ligament was located but left intact, with no transection performed. After surgery, the joint capsule and skin were sutured in layers using absorbable sutures. The surgical site was disinfected with povidone‐iodine, and the mice were placed on a heated pad for post‐operative recovery. The mice were closely monitored until full recovery of consciousness and mobility before being returned to their respective cages. Post‐operative care included daily observation for signs of discomfort or infection, and meloxicam was administered as needed for pain management.

### Preparation of DSPL NPs

DSPL NPs were prepared using a double emulsion solvent evaporation method performed as previously described.^[^ ^38^ ^]^ DS‐PLGA polymer materials were purchased from Xi'an Ruixi Biological Technology Co., Ltd. (Xi'an, China). Briefly, 5 mg of DS‐PLGA was dissolved in 300 µL ethyl acetate and 700 µL dichloromethane to form the organic phase. The compound Lut (MCE, HY‐N0162, USA), pre‐dissolved in dimethyl sulfoxide (DMSO), was carefully incorporated into the DS‐PLGA solution. The resulting mixture was subjected to ultrasonic treatment in a water bath for 5 min to achieve homogeneity. Subsequently, the acoustic probe was introduced into a pre‐prepared 3% polyvinyl alcohol (PVA, Beyotime, P105124, CHINA) solution, alternating between 50 W power output for 3 s and resting for 2 s during the emulsification process. The DS‐PLGA/Lut organic solvent mixture was gradually emulsified into the PVA solution, forming a water‐in‐oil emulsion. The emulsion was then transferred to a 0.3% PVA solution and stirred on a magnetic stirrer for 4 h to ensure complete evaporation of the organic solvents. After solvent removal, the resulting NPs were collected by centrifugation at 20000 × g for 20 min at 4 °C, washed with deionized water, and resuspended in PBS. The DSPL NPs were stored at 4 °C for further experiments.

### Preparation of MDSPL NPs

MDSPL was synthesized using a maleimide‐thiol coupling reaction. The thiol (‐SH) group from the **c**ysteine residue, which was artificially added during the commercial synthesis of the MIF‐mimetic peptide sequence (**C**LCGLLSDR), was covalently linked to the maleimide group of PLGA‐Se‐Se‐PEG‐Mal. To ensure the availability of free ‐SH groups, tris (2‐carboxyethyl) phosphine (TCEP) was added to the MIF peptide at a molar ratio of 1:1 and incubated at room temperature for 1 h. To facilitate covalent bonding between the maleimide terminal of DS‐PLGA and the thiol group from the cysteine residue, the MIF peptide/TCEP solution was mixed with the DSPL solution at a molar ratio of 1.5:1. The reaction was carried out in neutral PBS under gentle stirring, protected from light, overnight at room temperature. The MIF‐mimetic peptide functionalized MDSPL NPs were subsequently stored at 4 °C. As a control, DSPL NPs without MIF‐mimetic peptide conjugation were prepared following the same protocol but omitting the addition of the MIF peptide.

### Characterization of NPs

The particle size, zeta potential, and polydispersity index (PDI) of NPs at varying ratios were measured using a Zeta Sizer (Malvern, Worcestershire, UK) at room temperature. The stability of DSPL and MDSPL was evaluated by immersing the NPs in PBS for 1, 3, 5, 7, and 9 days. Changes in particle size and PDI were monitored using DLS with a Zetasizer Nano ZS (Malvern Instruments, Southborough, UK). The morphological characteristics of the NPs were observed via TEM, following previously established protocols.^[^ ^38^ ^]^ The drug loading capacity (DLC) and encapsulation efficiency (EE) were determined using a UV spectrophotometer to generate a standard curve. The calculations were performed using the following formulas:

(1) $$\mathrm{DLC}\left(\%\right)=\left(\left(\mathrm{Wi}-\mathrm{Ws}\right)/\mathrm{Wt}\right)\times 100$$

(2) $$\mathrm{EE}\left(\%\right)=\left(\left(\mathrm{Wi}-\mathrm{Ws}\right)/\mathrm{Wi}\right)\times 100$$

where Wi represents the initial drug input, Ws represents the drug content in the supernatant, and Wt represents the total weight of the NPs.

### Surface Plasmon Resonance

To examine the interaction between CD74 and Luteolin, CD74 was immobilized on Biacore CM5 sensor chips (Cytiva). The extracellular domain of CD74 protein, covering residues Gln73‐Met232, with an N‐terminal HA (YPYDVPDYA) tag was used. Coupling was performed with the Biacore amine coupling kit (Cytiva), which included NHS and EDC cross‐linkers, according to the manufacturer's protocol. In the binding experiment, coupling of CD74 resulted in a density of 6758 response units (RU) on the flow cell FC2. FC1 was used only to react with PBS running buffer. Luteolin was prepared using PBS, with the concentration set at 25, 12.5, 6.25, 3.125, and 0.75 µm. Each analyte (100 µL) was applied to the ligand‐coated chip at a flow rate of 30 µL min^−1^ for 3 min, using a BiacoreTM X100 Instrument (Cytiva). The dissociation time was 420 s and was followed by a 3 min regeneration with 100 µL of PBS

### Evaluation of Cellular Uptake and Localization of NPs

To assess the cellular uptake efficiency and intracellular localization of NPs in CD74^+^ macrophages, CLSM and FACS were utilized. Since Lut is non‐fluorescent, it was substituted with 1,1′‐dioctadecyl‐3,3,3′,3′‐tetramethylindodicarbocyanine, 4‐chlorobenzenesulfonate salt (DiD, Beyotime, C1039, CHINA), which was incorporated into the NPs. Cells were seeded in confocal dishes and six‐well plates, followed by incubation with NPs for specified time points (1, 3, 6, 9, and 12 h). After incubation, cells were fixed with 4% paraformaldehyde, permeabilized with 0.1% Triton X‐100 (Beyotime, P0096, CHINA), and stained with phalloidin‐Alexa Fluor 488 (Beyotime, C2201S, CHINA) for the cytoskeleton and DAPI for nuclei. Stained cells were visualized using a Zeiss LSM 880 confocal microscope (Germany). Quantitative fluorescence analysis was performed using a BD flow cytometer (USA) to determine cellular uptake levels.

### In Vivo Imaging for Biodistribution of NPs

To assess the biodistribution of NPs, 8‐week‐old mice treated with DMM surgery were randomly divided into three groups: DIR group, DSP‐DIR group, and MDSP‐DIR group. Different formulations were intra‐articularly injected into the knee joints, and retention time in the joint cavity was monitored. Fluorescence emission in the knee joint region was immediately measured using a small animal imaging system. At 24 h post‐injection, three mice from each group were euthanized, and blood, heart, liver, spleen, lung, kidney, stomach, and intestine samples were collected to evaluate fluorescence intensity using the imaging system. The remaining mice were monitored at 0, 1, 3, 7, 14, and 21 days post‐injection using the IVIS Lumina XRMS Series III imaging system (PerkinElmer, USA) to record fluorescence levels in the knee joint region. Quantitative analysis of fluorescence intensity from isolated tissues was performed using Living Image (64‐bit) software.

### ROS Detection Assay

ROS levels were measured following the manufacturer's protocol with minor modifications. Briefly, RAW 264.7 macrophages were seeded in confocal dishes at a density of 1 × 10⁶ cells per well. Cells were treated with different formulations and incubated with 2′,7′‐dichlorodihydrofluorescein diacetate (DCFH‐DA, 1:1000, Beyotime, S0033, CHINA) diluted in serum‐free medium for 20 min at 37 °C. Subsequently, cells were stained with Hoechst (Beyotime, C1027, CHINA) to visualize nuclei. ROS generation was detected using a laser confocal microscope. The fluorescent intensity of DCFH‐DA, indicative of intracellular ROS levels, was observed and quantified.

### Measurement of Mechanical Pain Sensitivity Using the Von Frey Test

Six mice were individually housed in separate cages with elevated wire mesh flooring. The animals were allowed a 20 min acclimation period to familiarize themselves with the environment, including the elevated platform. Mechanical sensitivity was assessed using Von Frey filaments applied perpendicularly to the plantar surface of the hind paw through the mesh floor. The filaments were gently pressed against the paw until they bent slightly, and the force was gradually increased. A withdrawal response, indicated by the mouse retracting its paw, was considered the threshold. The force required to elicit this response was recorded as the mechanical withdrawal threshold.

### Animal Model and Treatment Protocol

Eight‐week‐old male *C57BL/6J* mice were purchased from GemPharmatech (Nanjing, China). All animal experiments were conducted in compliance with guidelines approved by the local ethics committee. The specific methods of inducing the OA model through DMM surgery were detailed in the above‐mentioned methodology section. Mice were randomly assigned to the following treatment groups: Saline group (Control): IA injection of 10 µL saline; Lut group: IA injection of 10 µL Lut (30 µg mL^−1^) (Lut was initially dissolved in DMSO to prepare a 30 mg mL^−1^ stock solution. For IA injections, the stock solution was diluted in sterile physiological saline (0.9% NaCl) to achieve a final concentration of 30 µg mL^−1^ Lut with a residual DMSO concentration of 0.1% (v/v); PL group: IA injection of 10 µL DSPL NPs containing Lut (30 µg mL^−1^); MDSPL group: IA injection of 10 µL MDSPL NPs containing Lut (30 µg mL^−1^). The first injection was administered immediately after surgery, followed by injections every three weeks for a total duration of 12 weeks. All injections were performed using a 30‐gauge needle into the right knee joint (operated side). Behavioral analyses were conducted one week before sacrifice to assess functional outcomes. At 12 weeks post‐surgery, mice were euthanized, and knee joints and DRG tissues were collected for histological evaluation.

### Statistical Analysis

Statistical analyses were conducted using GraphPad Prism 8.0 (GraphPad Software). Data were expressed as the mean ± standard deviation (SD) or second quartile ± interquartile range (Q2 ± IQR), as specified in the figure legends. For comparisons between two groups, an unpaired, two‐tailed Student's t‐test was used for normally distributed data, while the Mann‐Whitney U test was applied for data that did not meet normality assumptions. When analyzing data across multiple groups, one‐way or two‐way analysis of variance (ANOVA) was performed, followed by appropriate post hoc tests, including Tukey's or Dunnett's test, depending on the experimental design. All experiments were independently repeated a minimum of three times, with details on the number of biological replicates provided in the corresponding figure legends. A *p*‐value < 0.05 was considered indicative of statistical significance.

## Conflict of Interest

The authors declare no conflict of interest.

## Author Contributions

R.P., B.Y., L.Z., and Z.W.X. contributed equally to this work. R.P., B.Y., L.Z., Z.W.X, Q.J.Y., Z.T.L., and S.Z.W. performed data curation, investigation, visualization, and wrote the original draft. Y.Q.H., H.J.W., H.Y.G., and S.W.H. performed investigation and visualization. R.P., B.Y., and Z.G.Z. performed formal analysis. T.J., X.F.Z., Y.L.W., and T.G. performed Supervision. T.G. performed conceptualization, supervision, wrote the original draft, and wrote, ‐reviewed, and edited the draft.

## Supporting information



Supporting Information

## Data Availability

The data that support the findings of this study are available from the corresponding author upon reasonable request.
