# Supplementary material for: Targeted Inhibition of CD74+ Macrophages by Luteolin via CEBPB/P65 Signaling Ameliorates Osteoarthritis Progression
Source: Adv Sci (Weinh). 2025 Nov 21;13(7):e08472. doi: 10.1002/advs.202508472 (PMC12866766; doi:10.1002/advs.202508472)

**Targeted Inhibition of CD74^+^ Macrophages by Luteolin via CEBPB/P65 Signaling Ameliorates Osteoarthritis Progression**

Rui Peng,^2^† Bo Yu,^3^† Lei Zhang,^5^† Zhaowen Xue,^4^† Lutian Yao,^6^ Qingjun Yang,^7^ Zitao Liu,^1^ Sizhi Wu,^8^ Yongquan Huang,^1^ Xiaofei Zheng,^4^ Huiying Guo,^10^ Songwei Huan,^10^ Tao Jiang,^1^***** Huajun Wang,^4^***** Yulong Wei,^9^***** Tao Gui^1^*****

^1^State Key Laboratory of Traditional Chinese Medicine Syndrome/Department of Orthopaedics, The Second Affiliated Hospital of Guangzhou University of Chinese Medicine, Guangzhou, Guangdong, China

^2^Department of Bone and Joint Surgery, The Affiliated Nanhua Hospital, Hengyang Medical School, University of South China, Hengyang, Hunan, China

^3^Department of Orthopedics, Medical Innovation Technology Transformation Center of Shenzhen Second People's Hospital, The First Affiliated Hospital of Shenzhen University, Shenzhen, China

^4^Department of Sports Medicine, The First Affiliated Hospital, Guangdong Provincial Key Laboratory of Speed Capability, The Guangzhou Key Laboratory of Precision Orthopedics and Regenerative Medicine, Jinan University, Guangzhou, Guangdong, China

^5^Department of General Surgery, The Second Affiliated Hospital of Bengbu Medical University, Bengbu, Anhui, China

^6^Department of Orthopaedics, The First Hospital of China Medical University, Shenyang, China

^7^College of Chinese Materia Medical, Tianjin University of Traditional Chinese Medicine, Tianjin, China

^8^Department of gerontology, Guangzhou First People's Hospital

^9^Department of Orthopaedics, Union Hospital, Tongji Medical College, Huazhong University of Science and Technology, Wuhan, China

^10^Department of Bone and Joint Surgery, The First Affiliated Hospital of Jinan University, Guangzhou, Guangdong, China

†These authors contributed equally to this work

Correspondence to:

Tao Jiang: [homejiangtao@126.com](mailto:homejiangtao@126.com); Huajun Wang: [hjwang@jnu.edu.cn](mailto:hjwang@jnu.edu.cn); Yulong Wei, [yulongwei@hust.edu.cn](mailto:yulongwei@hust.edu.cn); Tao Gui: [guitao@jnu.edu.cn](mailto:guitao@jnu.edu.cn)

Keywords: CD74, CEBPB, Macrophage, Luteolin, Osteoarthritis

**Supplementary data**


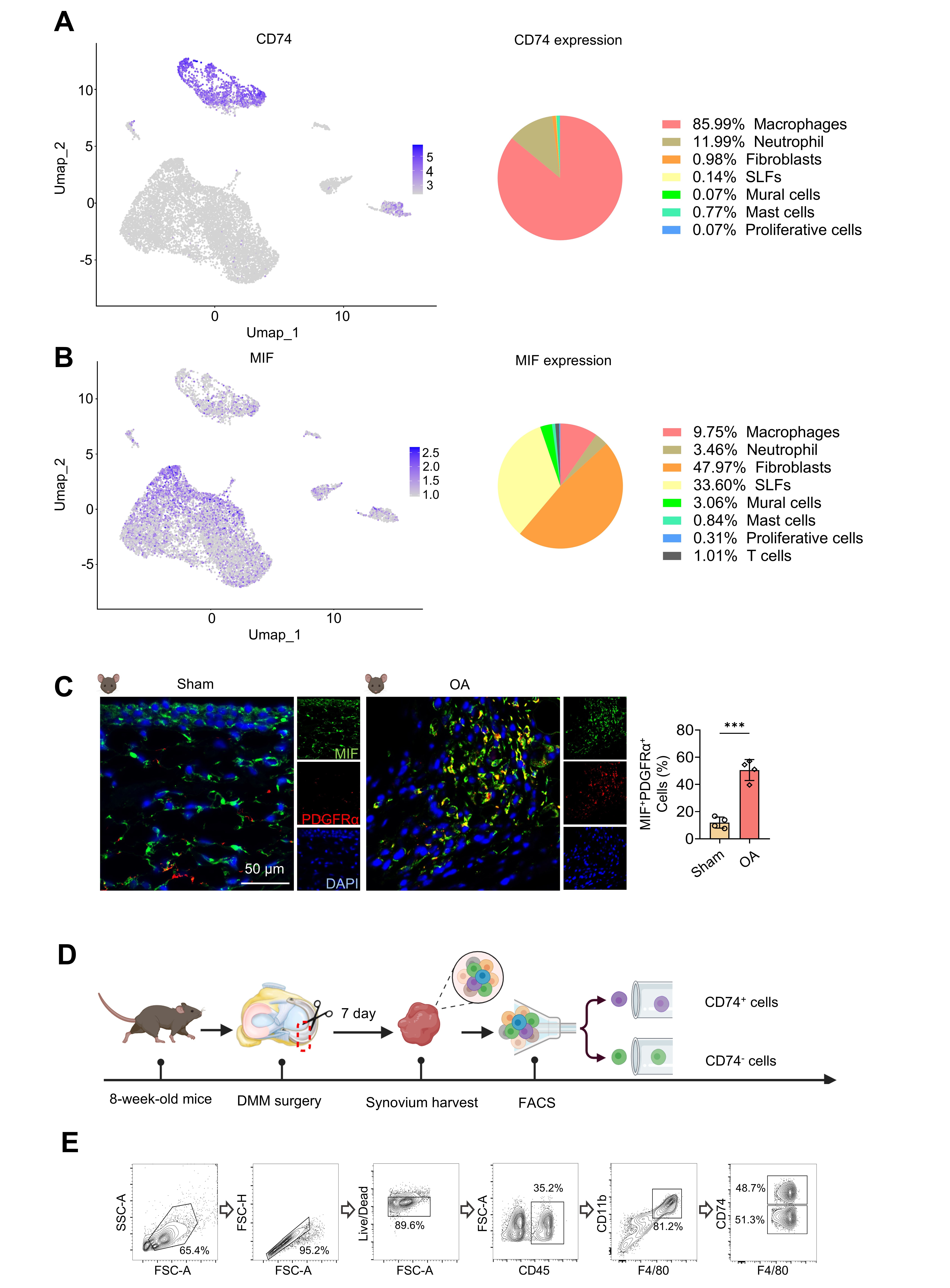


**Figure S1. Identification and characterization of CD74^+^ macrophage subpopulation in osteoarthritic synovial tissues.** A-B) The feature plot showing distribution of CD74 and MIF as well as their expression intensity across sub-populations. C) Left: Representative IF images of synovial tissues from Sham and DMM-induced OA mice, showing co-expression of MIF (green) with PDGFRα (red). Nuclei are visualized with DAPI (blue). Scale bar, 50 μm; Right: Quantitative assessment of MIF^+^PDGFRα^+^ cells. n = 4 independent biological replicates per group. D) Schematic diagram of primary synovial macrophages sorting of CD74^+^ and CD74^-^ macrophage populations from Sham and DMM-induced mouse inflammatory synovial tissues using FACS. E) The flow cytometry gating strategy to identify F4/80^+^CD74^+^ macrophage subsets. The data for all bar graphs is presented as mean ± SD, and P values were calculated by two-tailed unpaired Student's t-test. ****P* < 0.001.


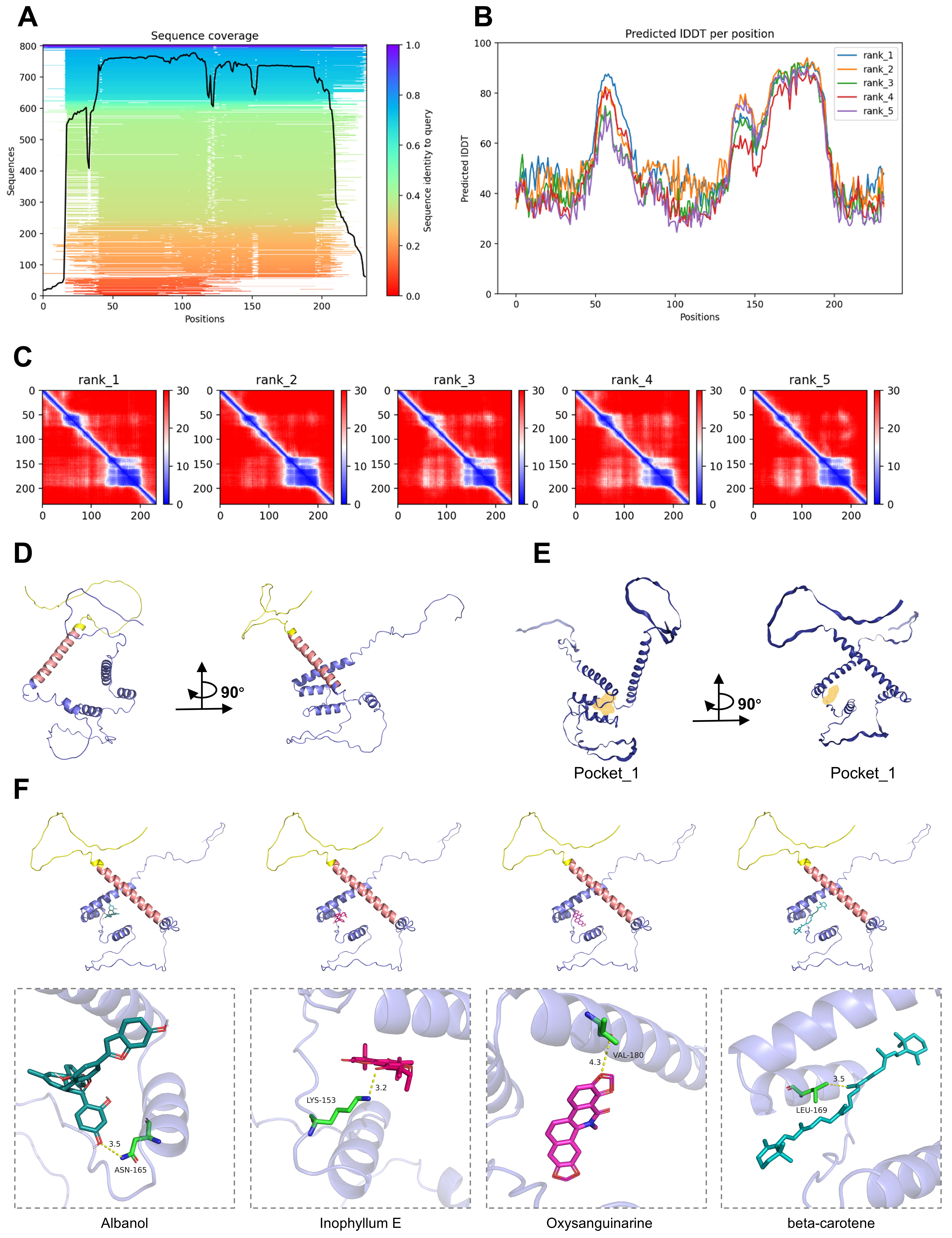


**Figure S2. Luteolin acts as a promising small-molecule agent in mediating the CD74-related macrophage inflammation process.** A) Peak plot delineating the predicted sequence coverage of the CD74 protein. B) Graphical representation of confidence indicators (plDDT) for CD74 protein pocket prediction. C) Predicted Aligned Error (PAE) matrix for quantitative assessment of CD74 protein structural prediction errors. D) Three-dimensional structure of the CD74 protein consisting of the intracellular region, transmembrane region, and extracellular segment. E) Visualization of pocket_1 as the protein pocket for virtual screening. F) Visualization analysis of molecular docking on the top 5 molecules binding with the intracellular region of the CD74 protein.


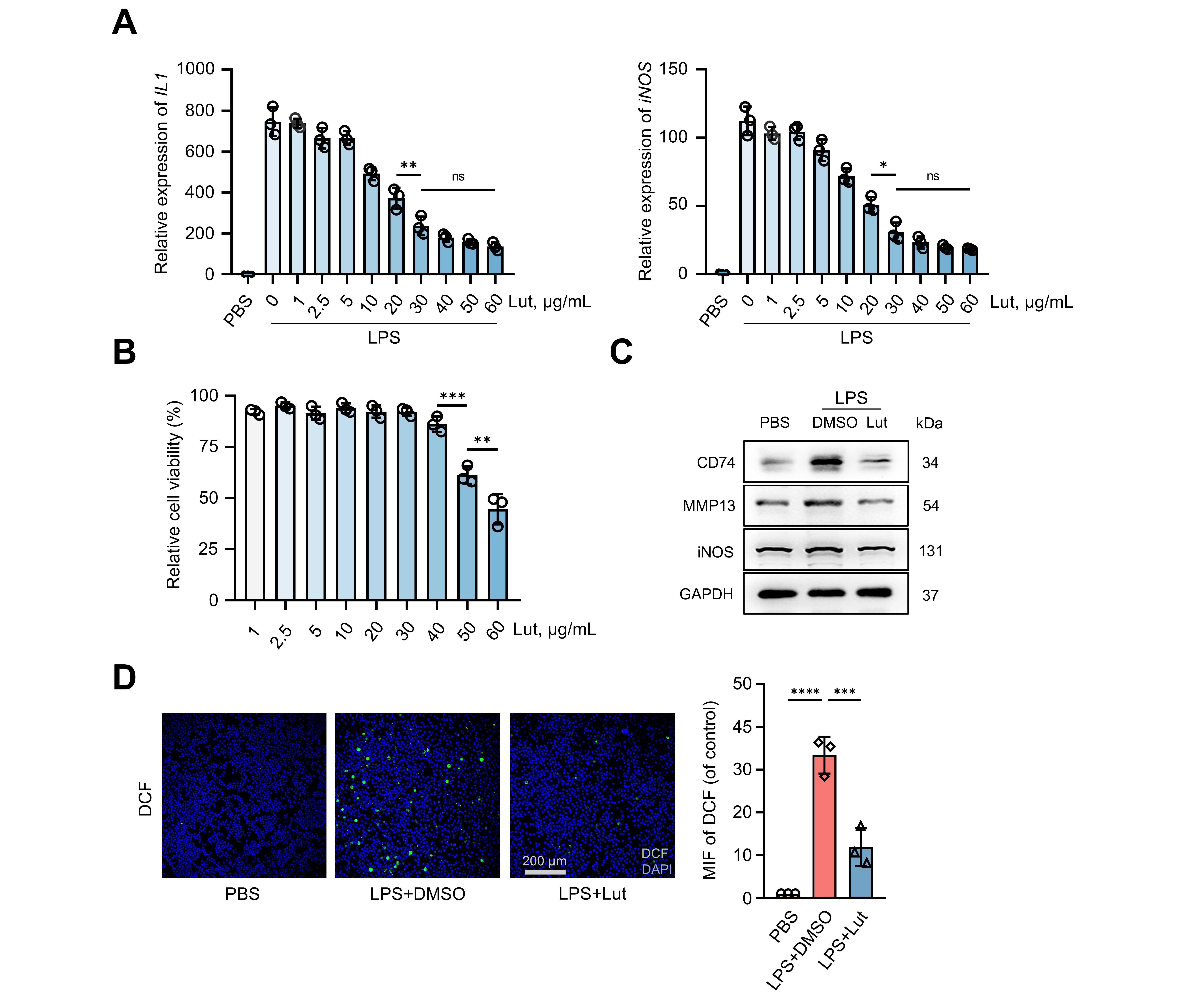


**Figure S3. Luteolin acts as a promising small molecule agent in mediating the CD74-related macrophage inflammation process.** A) The mRNA expression levels of *IL1* and *iNOS* in macrophages treated with varying concentrations of Lut under LPS stimulation (n = 3 independent biological replicates per group). B) The relative cell viability of macrophages under different concentrations of Lut (n = 3 independent biological replicates per group). C) IB analysis of CD74, iNOS and MMP13 in LPS-stimulated RAW 264.7 cells with or without Lut treatment. D) IF detection and quantitative statistics of ROS intensity in RAW 264.7 cells treated with DMSO or Lut with or without LPS stimulation. The data are presented as mean ± SD. ns: not significant, **P* < 0.05, ***P* < 0.01, ****P* < 0.001.


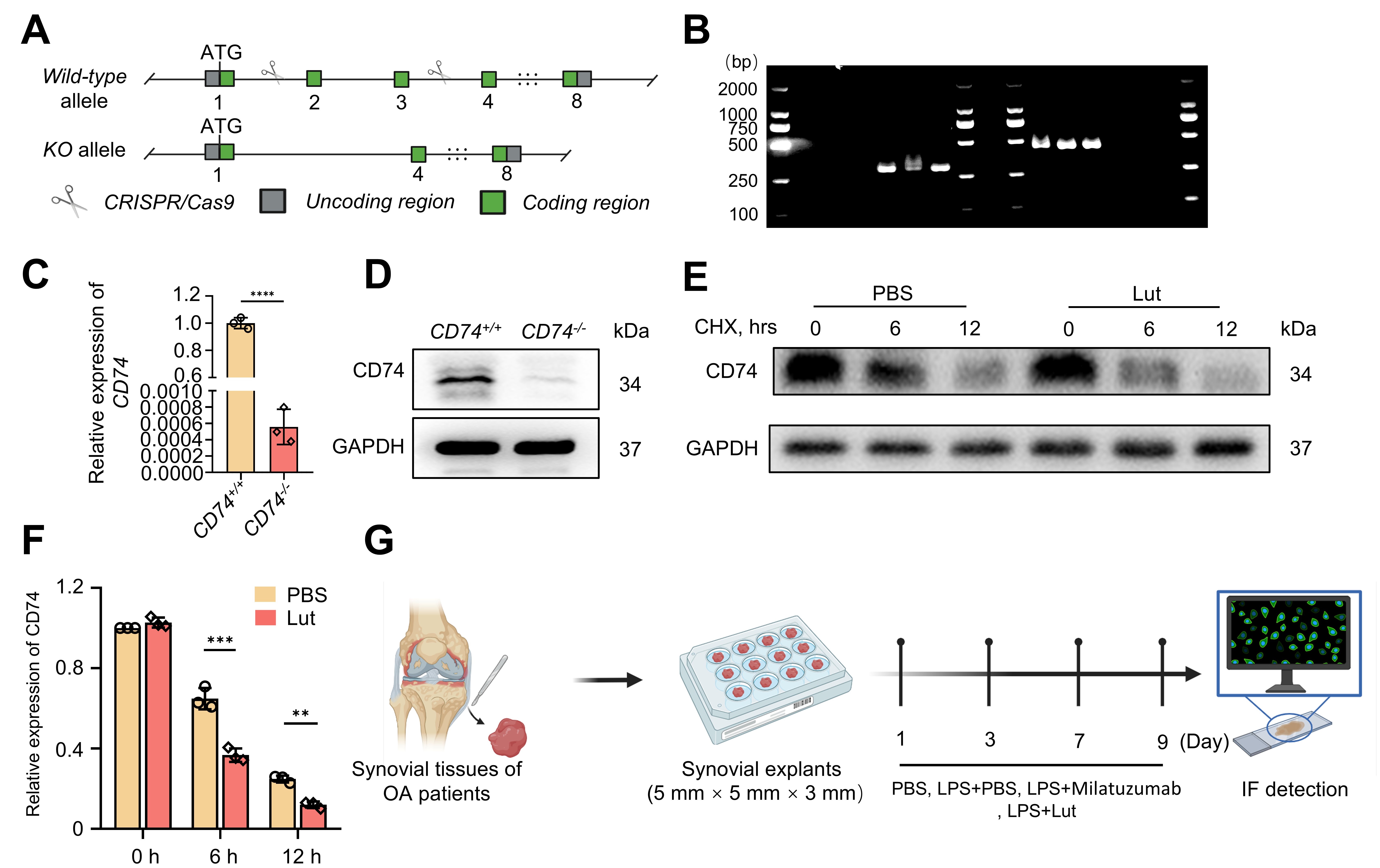


**Figure S4. Luteolin acts as a promising small molecule agent in mediating the CD74-related macrophage inflammation process.** A) The schematic diagram using CRISPR/Cas9 technology to edit the CD74 gene. B) Genotyping of *CD74^-/-^* by gel electrophoresis. C) Relative mRNA expression detection of *CD74* in *CD74^+/+^-* and *CD74^-/-^-*derived primary mBMDMs by qPCR (n = 3 independent biological replicates per group). D) IB analysis detecting the protein expression level of CD74 in *CD74^+/+^-* and *CD74^-/-^-*derived primary mBMDMs. E) Cycloheximide (CHX) chase assays showed that Lut accelerated the degradation of CD74 protein in RAW 264.7 cells, as evidenced at 0, 6, and 12 h. F) Quantitative densitometric analysis of CD74 protein band intensity in PBS- and Lut-treated group. G) Schematic diagram of *ex vivo* human synovial explants isolation, culture and lut treatment strategy. The data are presented as mean ± SD. P values were calculated by (C) two-tailed unpaired Student's t-test and (F) two-way ANOVA. ***P* < 0.01, ****P* < 0.001, *****P* < 0.0001.


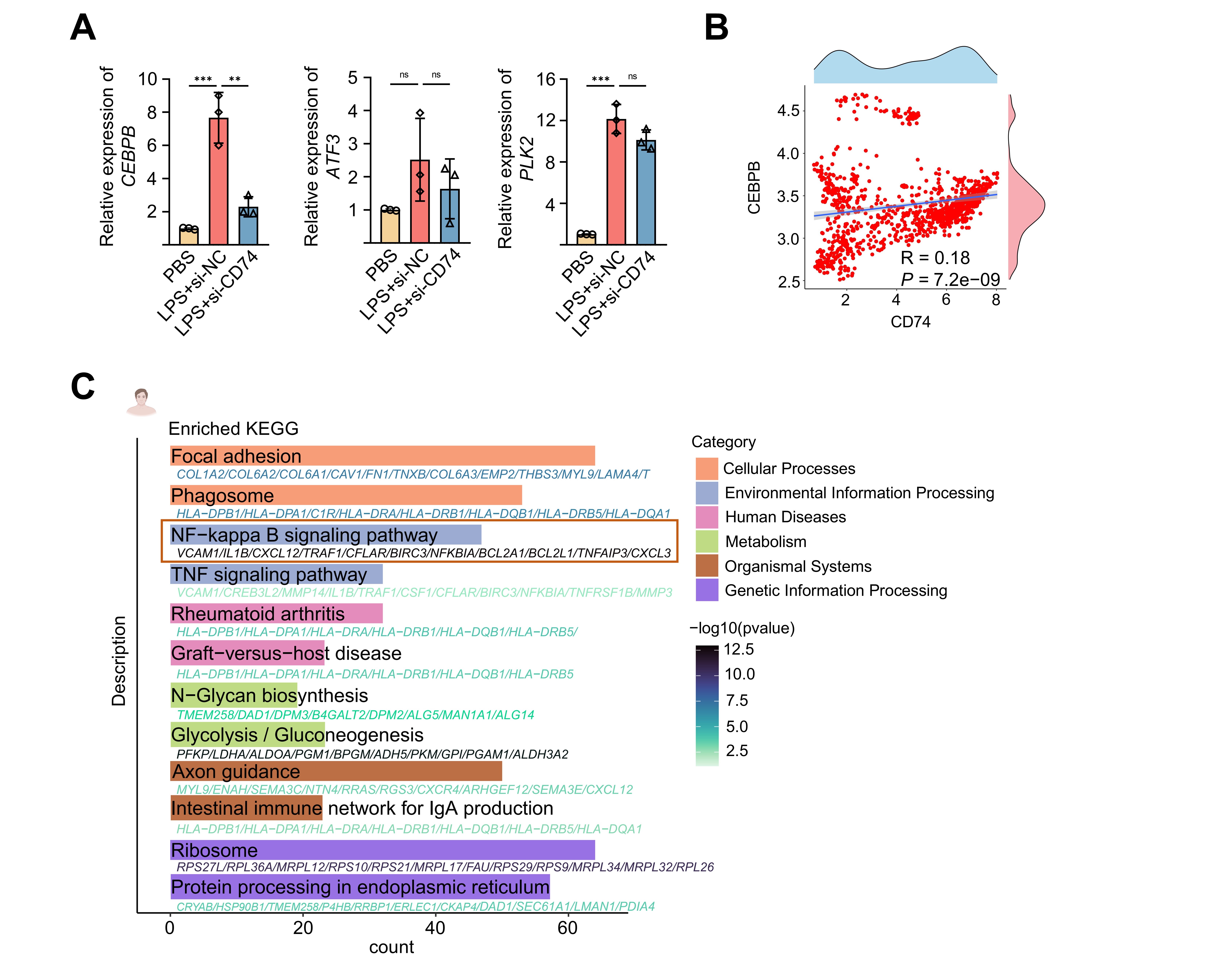


**Figure S5. Luteolin attenuates macrophage-mediated inflammation via targeting the CD74/CEBPB/P65 axis.** A) Relative mRNA expression detection of *CEBPB*, *ATF3,* and *PLK2* in *CD74^+/+^-* and *CD74^-/-^-*derived primary mBMDMs with or without LPS activation (n = 3 independent biological replicates per group). B) Pearson correlation analysis between CD74 and CEBPB in CD74^high^ macrophages from synovial tissues of mice (7 days after the DMM modeling surgery). C) The KEGG enrichment analysis comparing Cluster C1 macrophage subpopulations of synovial tissues from OA patients with those from Non-OA groups. The data are presented as mean ± SD. P values were calculated by one-way ANOVA. ns: not significant. ***P* < 0.01, ****P* < 0.001.


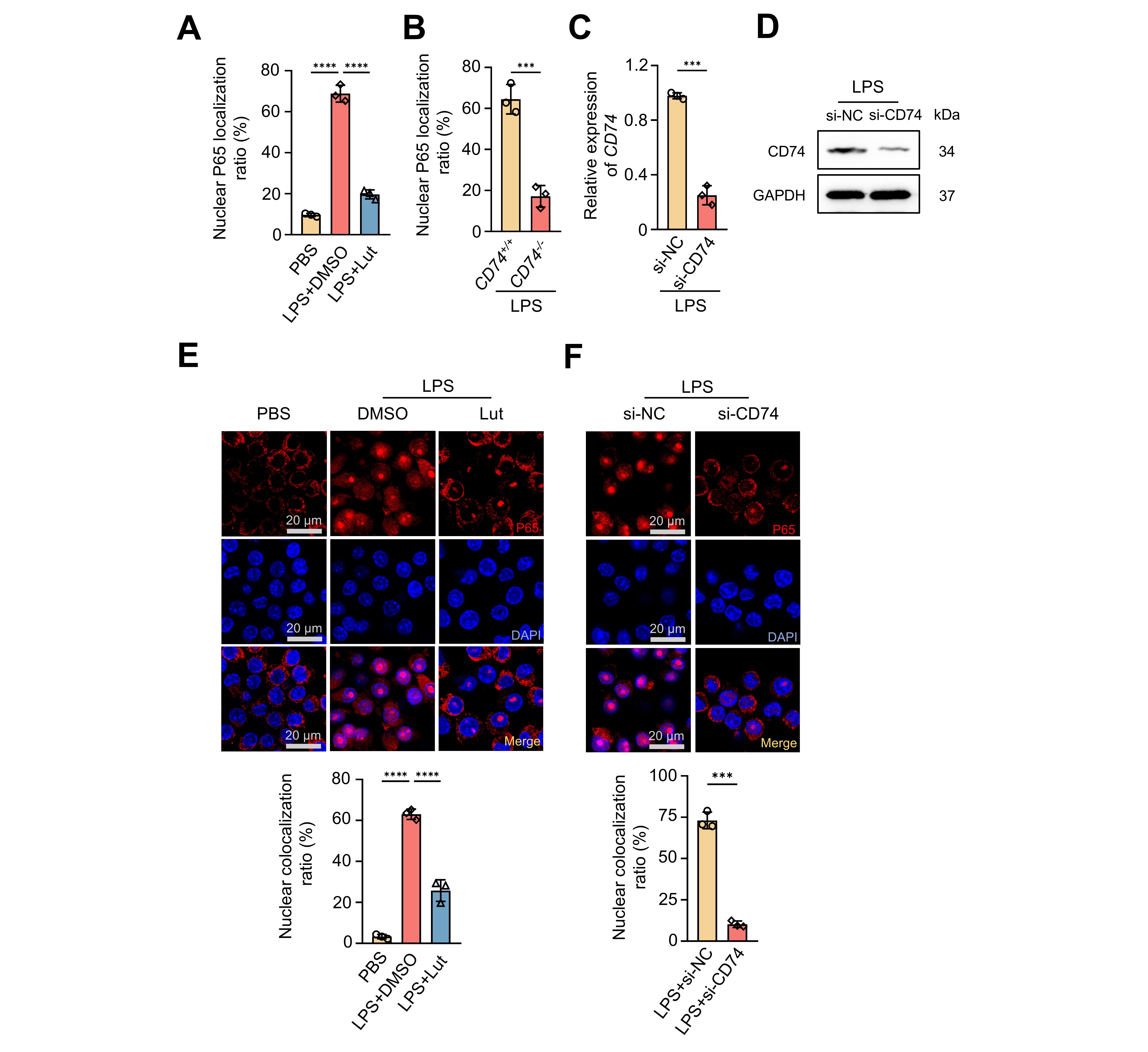


**Figure S6. Luteolin inhibits macrophage inflammatory response through the CD74/CEBPB/P65 axis.** A) Quantitative assessment of P65 nuclear colocalization ratio in primary mBMDMs activated by LPS with or without Lut treatment (n = 3 independent biological replicates per group). B) Quantitative assessment of P65 nuclear colocalization ratio in *CD74^+/+^* and *CD74^-/-^* primary mBMDMs activated by LPS (n = 3 independent biological replicates per group). C-D) Transcriptional and translational detection of CD74 level in *CD74^+/+^* and *CD74^-/-^-*derived mBMDMs (n = 3 independent biological replicates per group). E) Upper panel: Representative fluorescence images showing p65 localization in RAW 264.7 cells activated by LPS with or without Lut treatment. Lower panel: Quantitative assessment of P65 nuclear colocalization ratio in RAW 264.7 cells (n = 3 independent biological replicates per group). Scale bar: 20 μm. F) Upper panel: Representative fluorescence images showing p65 localization in RAW 264.7 cells activated by LPS, transfected with or without si-CD74. Lower panel: Quantitative assessment of P65 nuclear colocalization ratio in RAW 264.7 cells with or without CD74 knockdown (n = 3 independent biological replicates per group). Scale bar: 20 μm. P values were calculated by (A and E) one-way ANOVA and (B-C and F) two-tailed unpaired Student's t-test. ****P* < 0.001, *****P* < 0.0001.


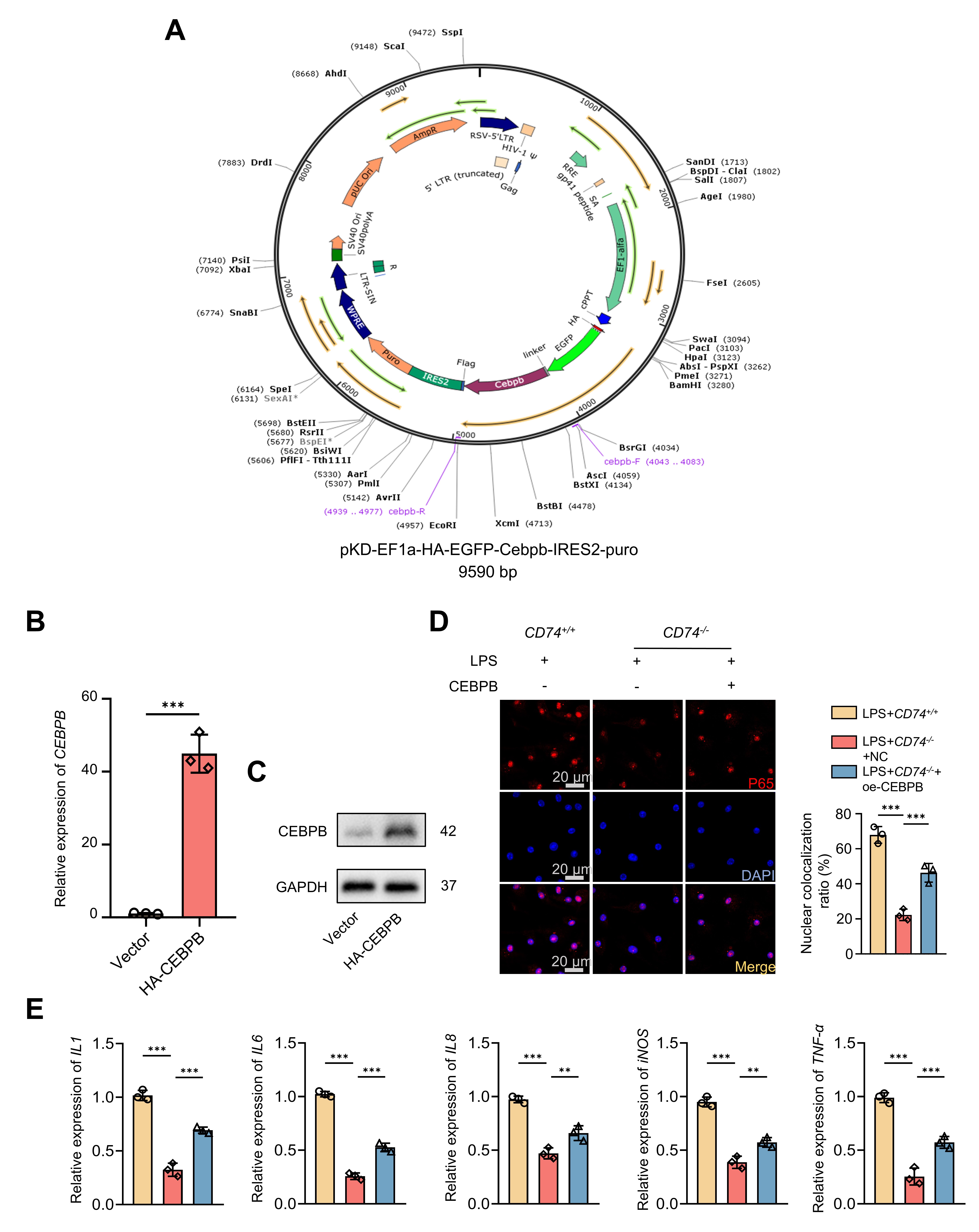


**Figure S7. Luteolin inhibits macrophage inflammatory response through the CD74/CEBPB/P65 axis.** A) Schematic representation of the plasmid construction encoding the CEBPB protein. B-C) Overexpression of CEBPB in RAW 264.7 cells was validated by quantifying both mRNA and protein expression levels using qRT-PCR and Western blot analyses, respectively. D) Representative fluorescence images showing p65 localization in *CD74^+/+^* and *CD74^-/-^*-derived primary mBMDMs transfected with or without CEBPB-overexpression plasmids in the background of LPS activation (n = 3 independent biological replicates per group). Scale bar: 20 μm. E) Relative mRNA expression detection of *IL1*, *IL6*, *IL8*, *iNOS,* and *TNF-α* in *CD74^+/+^* and *CD74^-/-^-*derived primary mBMDMs transfected with or without CEBPB-overexpression plasmids in the background of LPS activation (n = 3 independent biological replicates per group). P values were calculated by (D and E) one-way ANOVA and (B) two-tailed unpaired Student's t-test. ***P* < 0.01, ****P* < 0.001.


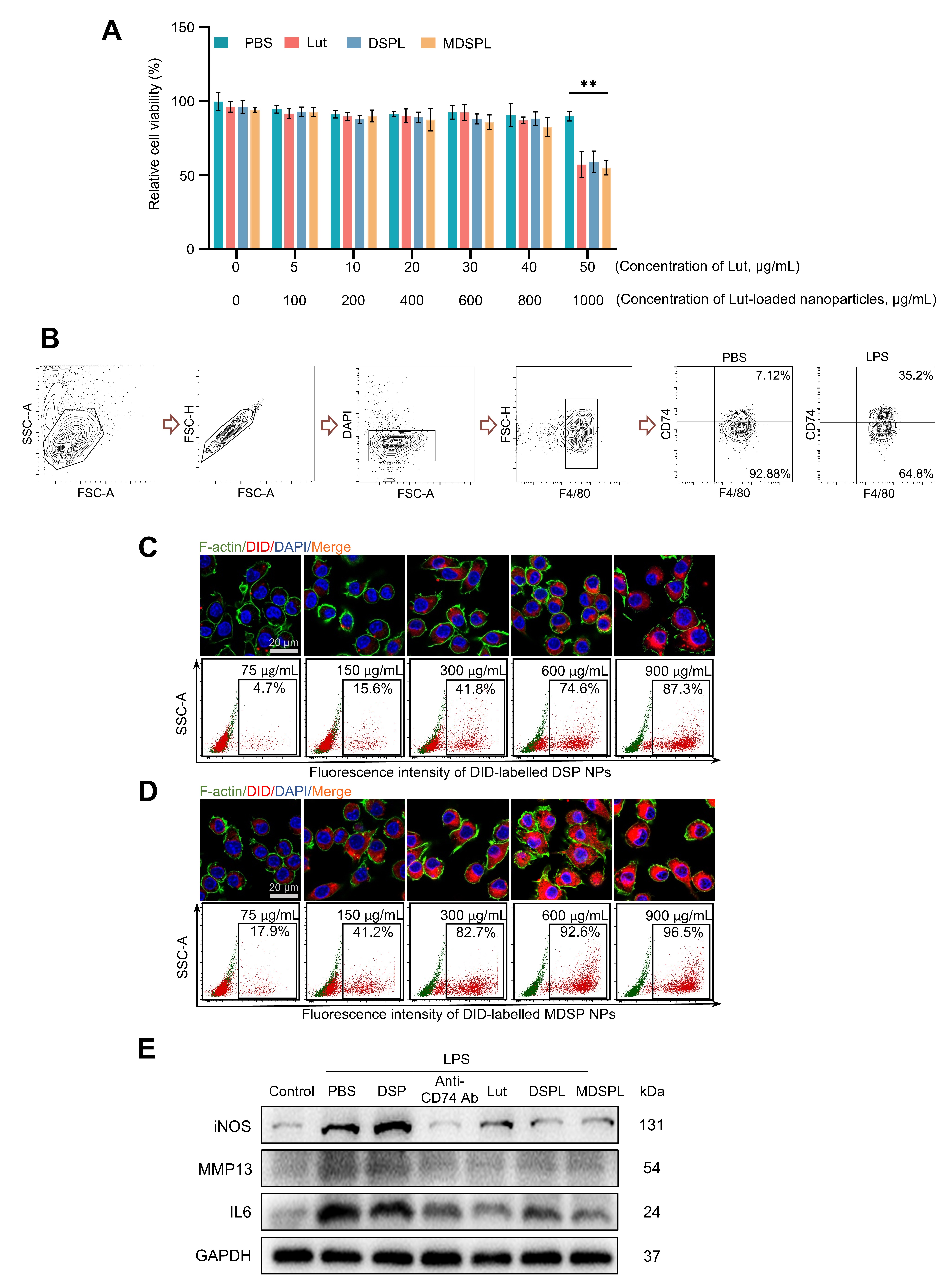


**Figure S8. MDSPL NPs exhibit enhanced cellular uptake and exert anti-inflammatory effects in CD74^+^ macrophages.** A) The relative cell viability of LPS-activated RAW 264.7 cells under different concentrations of Lut and Lut-containing NPs (n = 3 independent biological replicates per group). B) The flow cytometry gating strategy to identify F4/80^+^CD74^+^ macrophage subsets in LPS-activated RAW 264.7 cells. C-D) Concentration-dependent cellular uptake of DSP-DiD and MDSP-DiD NPs assessed by CLSM (upper panel) and FACS (lower panel). CD74^+^ macrophages were simultaneously treated with DSP-DiD and MDSP-DiD NPs at varying concentrations (75 μg/ml, 150 μg/ml, 300 μg/ml, 600 μg/ml, 900 μg/ml, respectively). Scale bar: 20 μm. E) IB analysis of iNOS, MMP13 and IL6 in RAW 264.7 cells treated by Lut and different formulations of NPs with or without LPS stimulation. The data are presented as mean ± SD. P values were calculated by (A) two-way ANOVA. ***P* < 0.01.


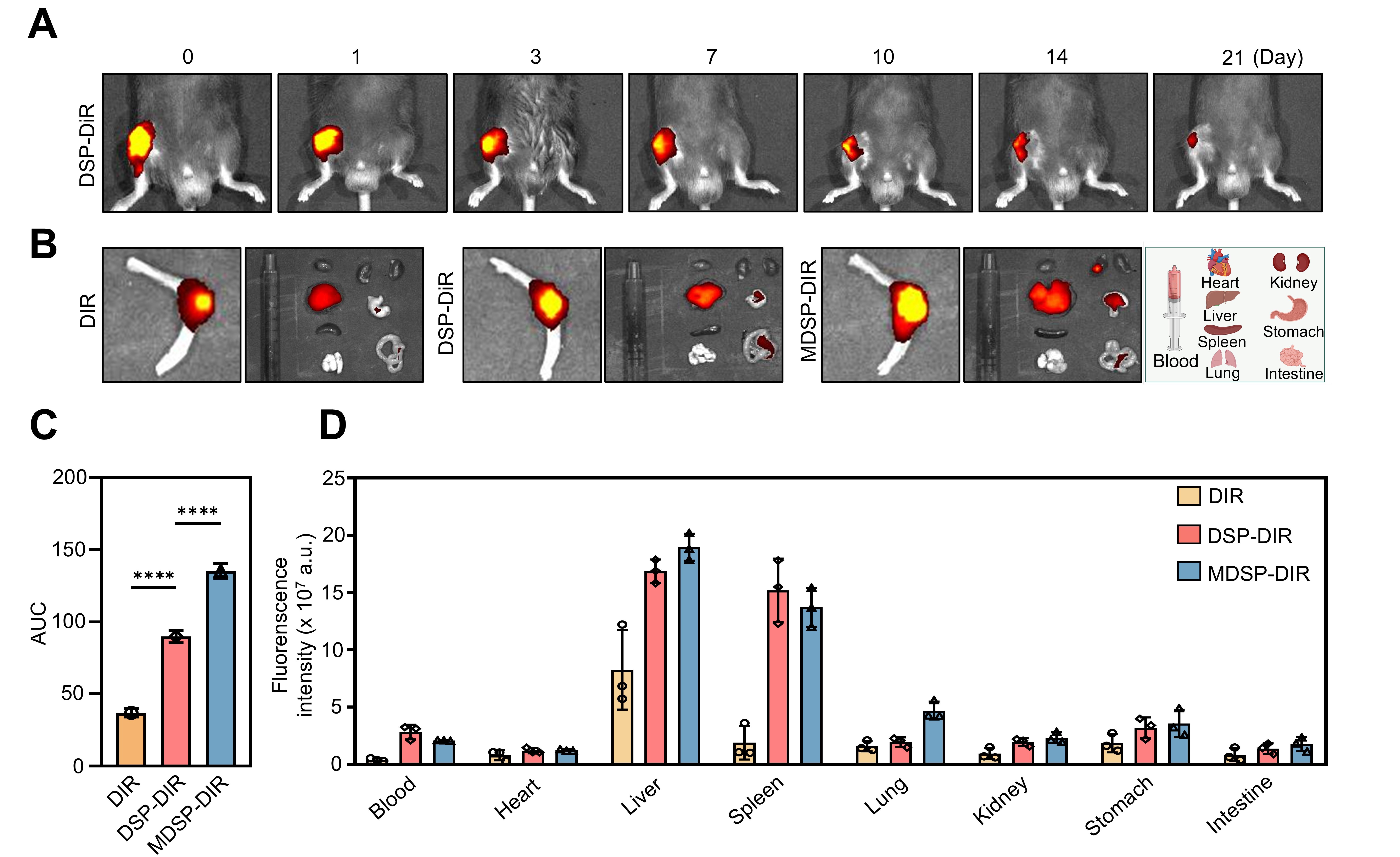


**Figure S9. MDSPL NPs exhibit enhanced cellular uptake and exert anti-inflammatory effects in CD74^+^ macrophages.** A) Fluorescence localization detection in joints of mice at different time points after IA injection of DSP-DIR NPs. B) Systematic biodistribution studies conducted 24 h post-injection demonstrated the tissue-specific accumulation patterns of DiR-labeled NPs across the knee-joint and major organ systems (cardiac, hepatic, splenic, pulmonary, renal, and gastrointestinal tissues) and blood samples. C) Quantification of area under fluorescence intensity/time curve of different nano groups (n = 3 independent biological replicates per group). D) Quantitative analysis of tissue-specific fluorescence intensity of blood and major organ systems (cardiac, hepatic, splenic, pulmonary, renal, and gastrointestinal tissues) 24 h after injection of DiR-labeled NPs (n = 3 independent biological replicates per group). The data are presented as mean ± SD. P values were calculated by (C) one-way ANOVA. *****P* < 0.0001.


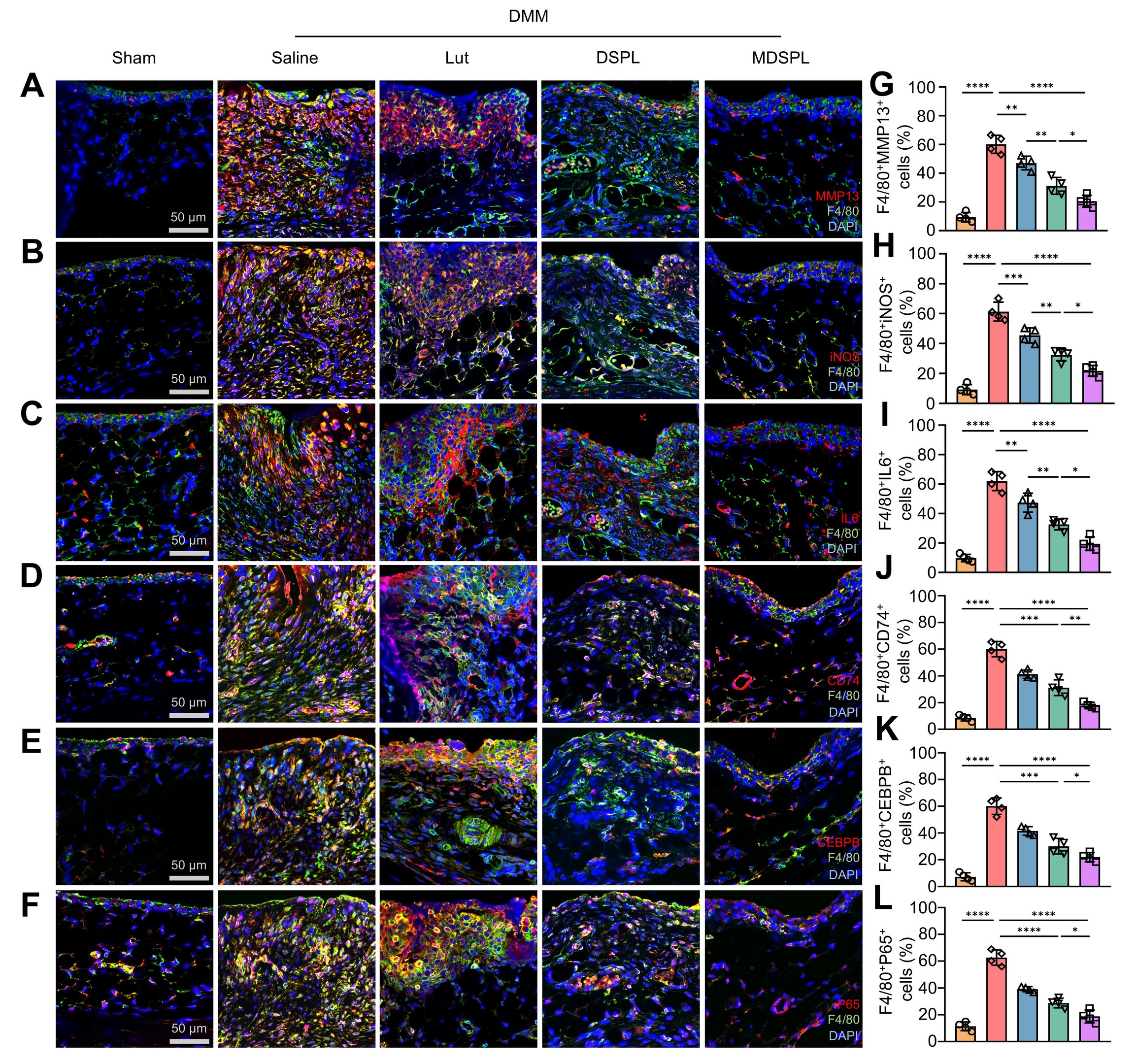


**Figure S10. MDSPL NPs alleviate OA-associated pain and cartilage damage *in vivo.*** A-C) Representative fluorescence images showing MMP13, iNOS, and IL6 co-localization with F4/80 in synovial tissues of the Sham and DMM group (12 weeks after the DMM modeling surgery). Scale bar: 50 μm; G-I) Quantitative assessment showing MMP13, iNOS, and IL6 co-localization percentage with F4/80 in synovial tissues from the mouse treated with different treatments (n = 4 independent biological replicates per group). D-F) Representative fluorescence images showing CD74, CEBPB, and P65 co-localization with F4/80 in synovial tissues of the Sham and DMM group (12 weeks after the DMM modeling surgery). Scale bar: 50 μm; J-L) Quantitative assessment showing CD74, CEBPB, and P65 co-localization percentage with F4/80 in synovial tissues from the mouse treated with different treatments (n = 4 independent biological replicates per group). The data are presented as mean ± SD. P values were calculated by (G-L) one-way ANOVA. **P* < 0.05, ***P* < 0.01, ****P* < 0.001, *****P* < 0.0001.


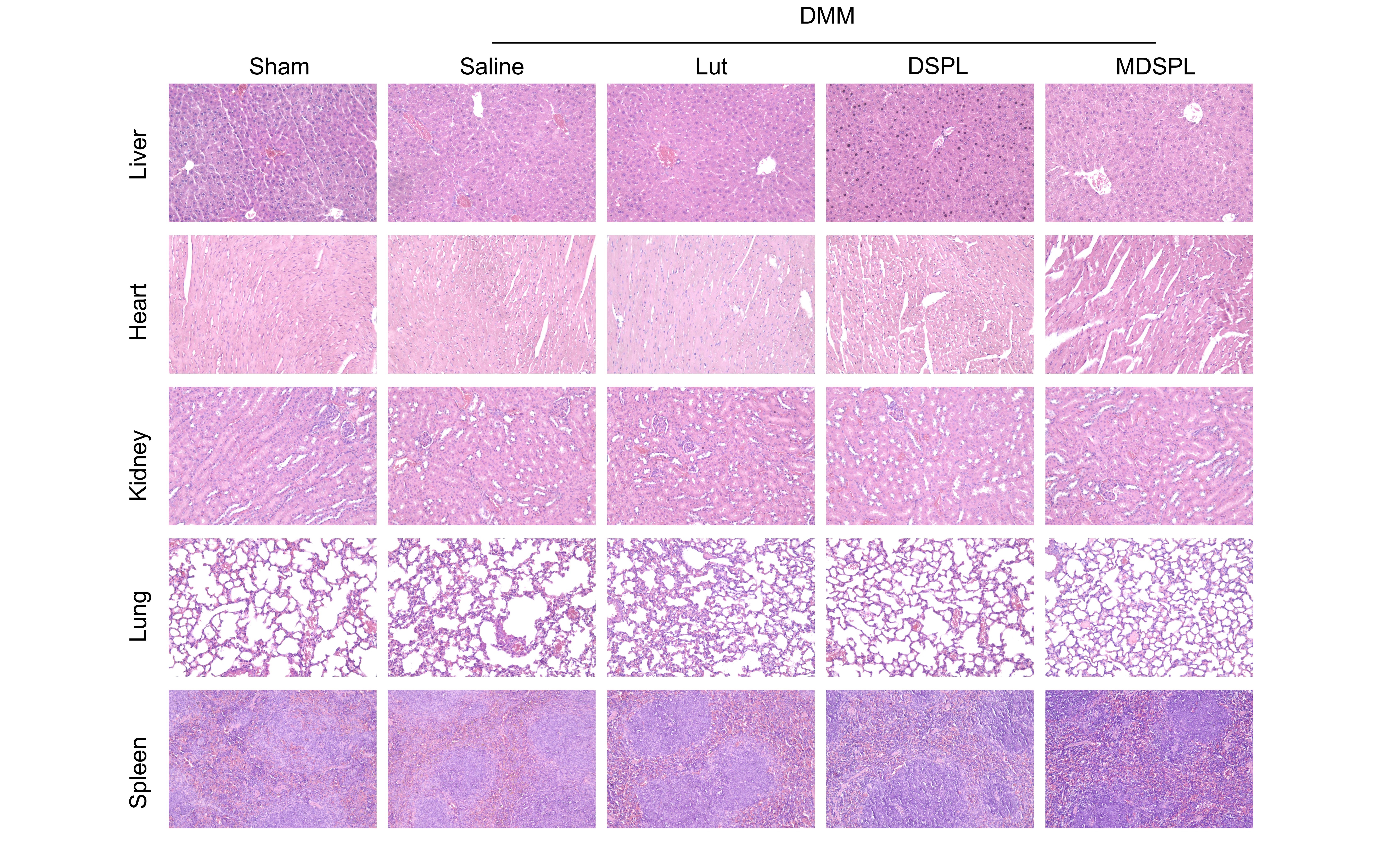


**Figure S11.** ***In vivo* evaluation of biosafety**. HE staining of heart, liver, spleen, lung, and kidney tissues from Sham and by Lut and different formulations of NPs treatment groups.


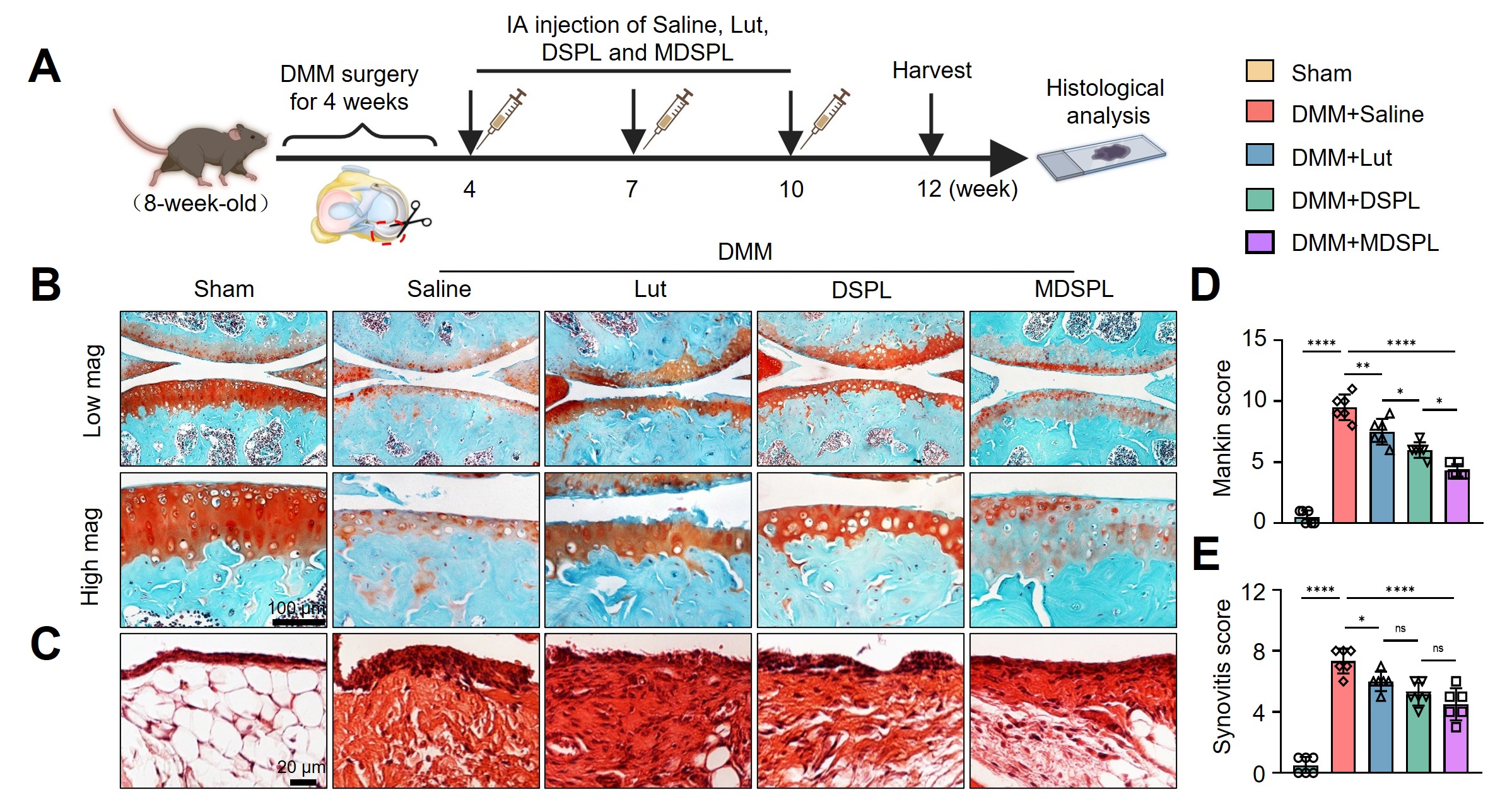


**Figure S12. MDSPL NPs alleviate OA-associated pain and cartilage damage *in vivo.*** A) Schematic diagram illustrating the establishment of the OA mice model and the therapeutic evaluation of indicated treatments. B-C) Representative Safranin O/Fast green (Scale bar: 100 μm) and HE staining images (Scale bar: 20 μm) of knee joints from Sham and by Lut and different formulations of NPs treatment groups 12 weeks after the DMM modeling surgery. D-E) Quantitative assessment of OA severity with Mankin scores and synovial scores from Sham and by Lut and different formulations of NPs treatment groups (n = 6 independent biological replicates per group). The data are presented as mean ± SD. P values were calculated by (D-E) one-way ANOVA. n.s.: not significant, **P* < 0.05, ***P* < 0.01, *****P* < 0.0001.

**Table S1:** Pocket information for the structure of CD74 protein.


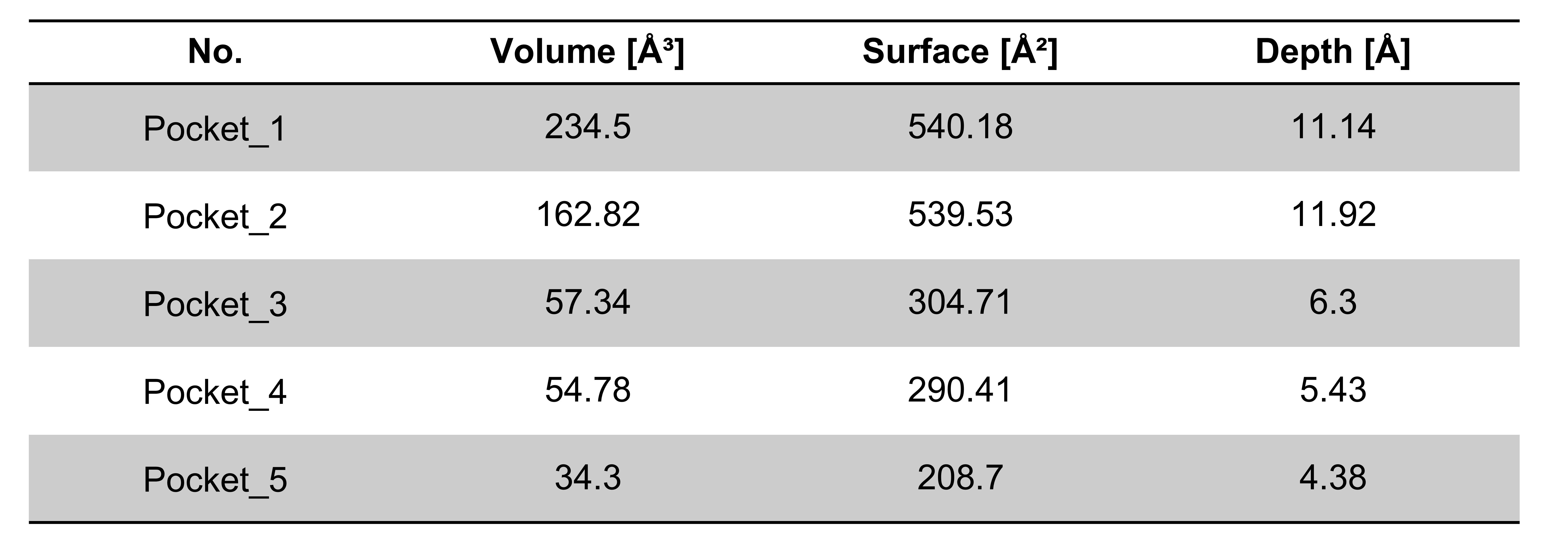


**Table S2:** Docking information for network pharmacology analysis.


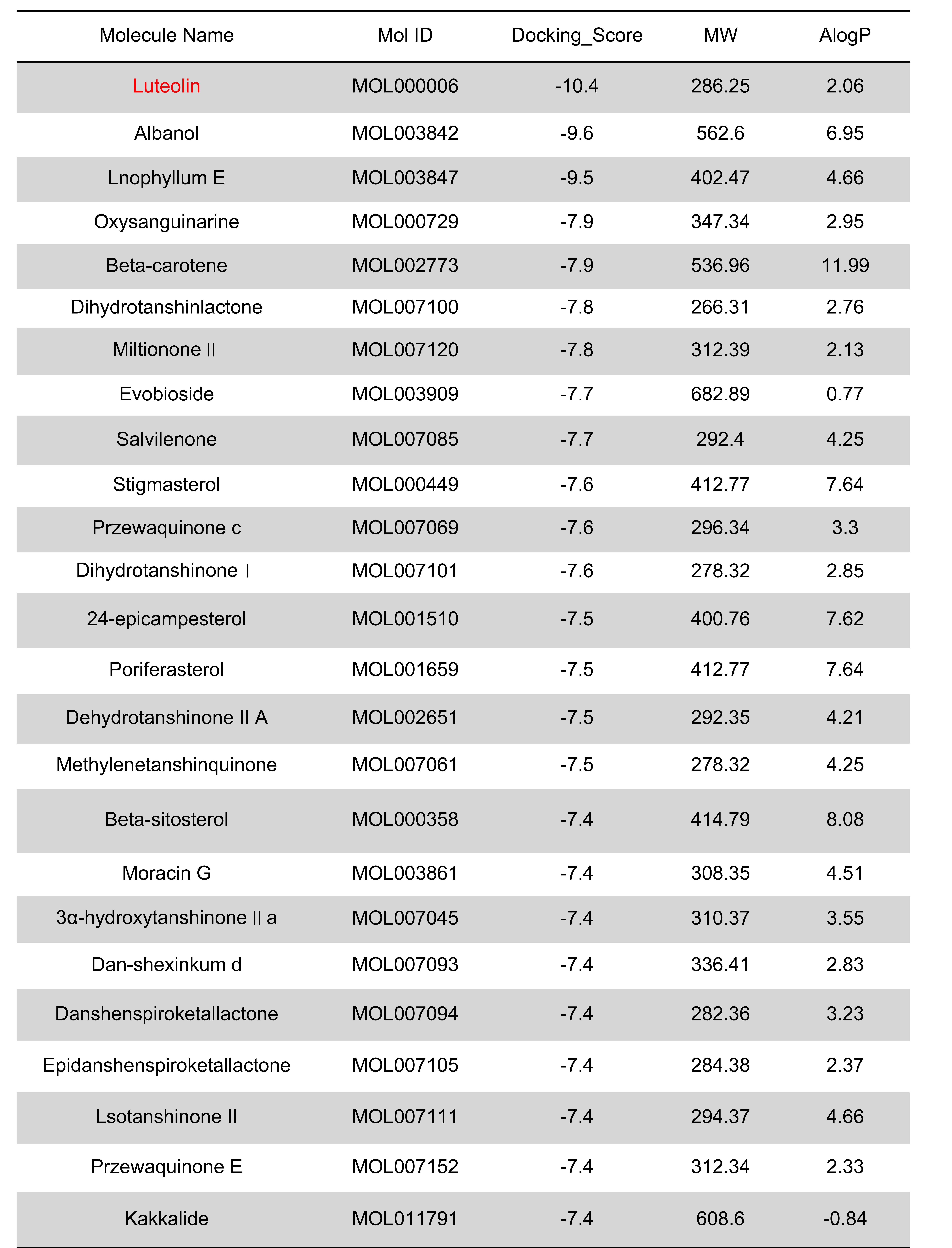


**Table S3:** The sequence of siRNAs used in this study.


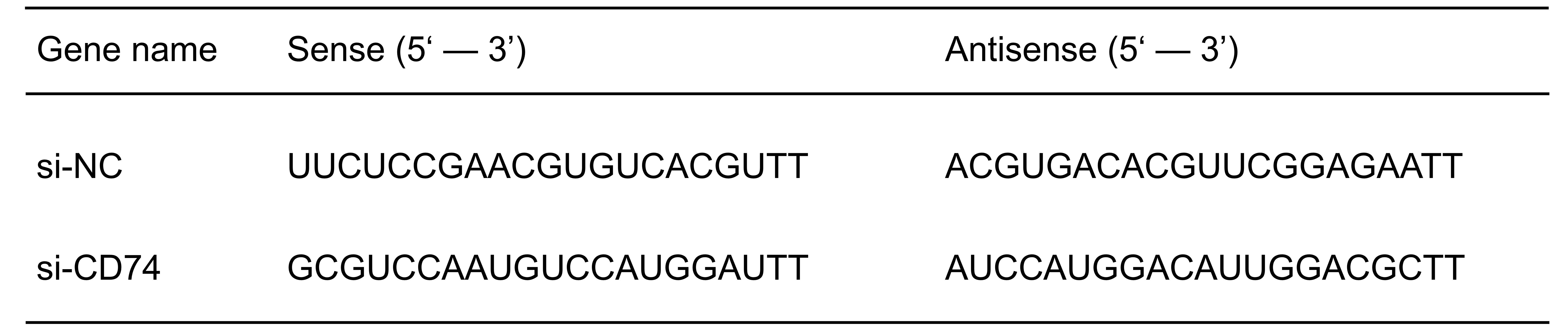


**Table S4** PCR primers used to construct plasmids.


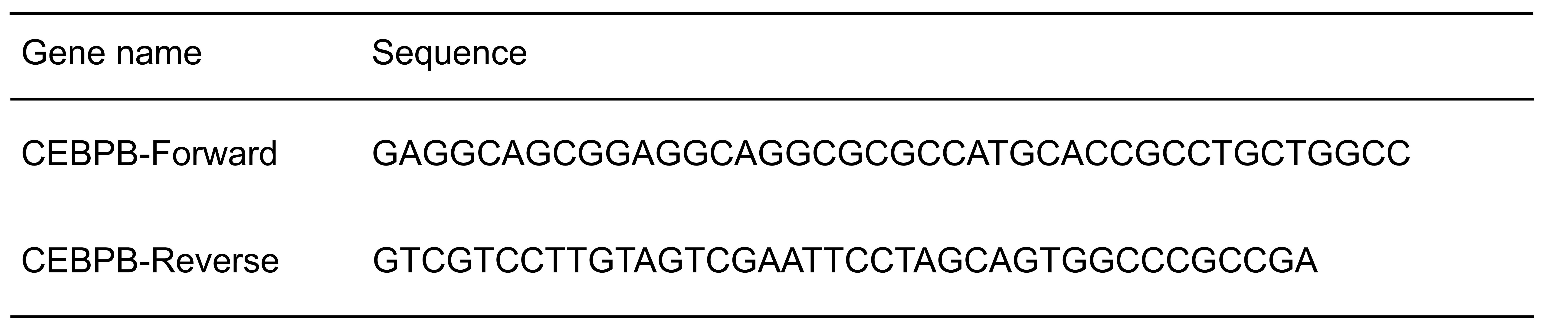


**Table S5:** qPCR primers used in this study.


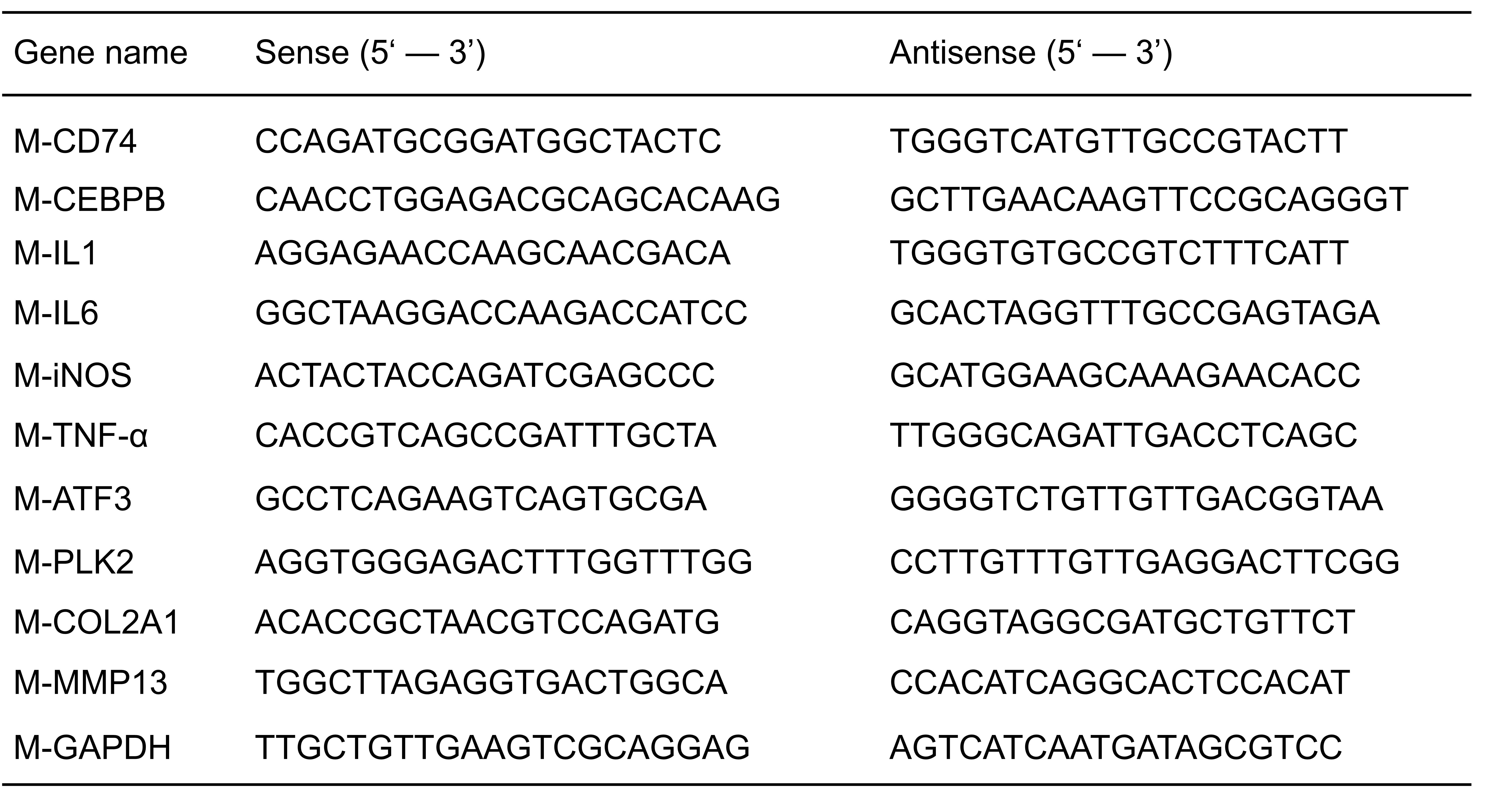

Supplement: Supplementary file 1 — Supporting Information [file ADVS-13-e08472-s001.docx]
